# Conditionally Significant eQTL and TWAS Analyses Identify MsRD26 as a Major Candidate Positive Regulator of Salt Tolerance in Medicago sativa L

**DOI:** 10.1002/advs.77098

**Published:** 2026-08-11

**Authors:** Lin Chen, Yuqi Zhang, Xinyue Ma, Jinpeng Bi, Fei He, Li Zhao, Haiyue Lei, Zhengqin Xiao, Xue Wang, Tiejun Zhang, Ruicai Long, Junmei Kang, Qingchuan Yang

**Affiliations:** ^1^ Institute of Animal Science Chinese Academy of Agricultural Sciences Beijing China; ^2^ State Key Laboratory of Vegetable Biobreeding Institute of Vegetables and Flowers Chinese Academy of Agricultural Sciences Beijing China

**Keywords:** alfalfa, candidate gene, eQTL, MsRD26, salt tolerance

## Abstract

Natural variation in gene expression bridges genetic polymorphisms and phenotypic divergence, yet the regulatory architecture underlying salt stress responses remains largely unexplored in alfalfa. Here, we generated 528 RNA‐seq libraries from 176 alfalfa accessions under well‐watered and salt stress conditions and identified 12,901 differentially expressed genes. Through integration of population transcriptomics, eQTL mapping, TWAS, and Mendelian randomization, we constructed a genome‐wide regulatory landscape comprising 62,423 to 67,405 eQTLs, 346 distant eQTL hotspots, and a predictive TF–eGene interaction network. We prioritized 1,401 genes whose expression variations are associated with salt tolerance. Among these, we functionally validated *MsRD26*, a NAC transcription factor, as a major candidate positive regulator of salt tolerance. This study provides a comprehensive resource of regulatory variants and candidate genes for salt tolerance in alfalfa, with implications for genomics‐assisted breeding in this polyploid forage crop.

## Graphical abstract

Salt stress significantly impacts plant growth. Through population transcriptome, eQTL and TWAS analyses, we identified several candidate genes potentially associated with salt stress response. Functional assays suggested that some of these genes may contribute to salt tolerance. These findings provide insights into the mechanisms underlying alfalfa's adaptation to salt stress and offer clues for breeding stress‐tolerant crops.

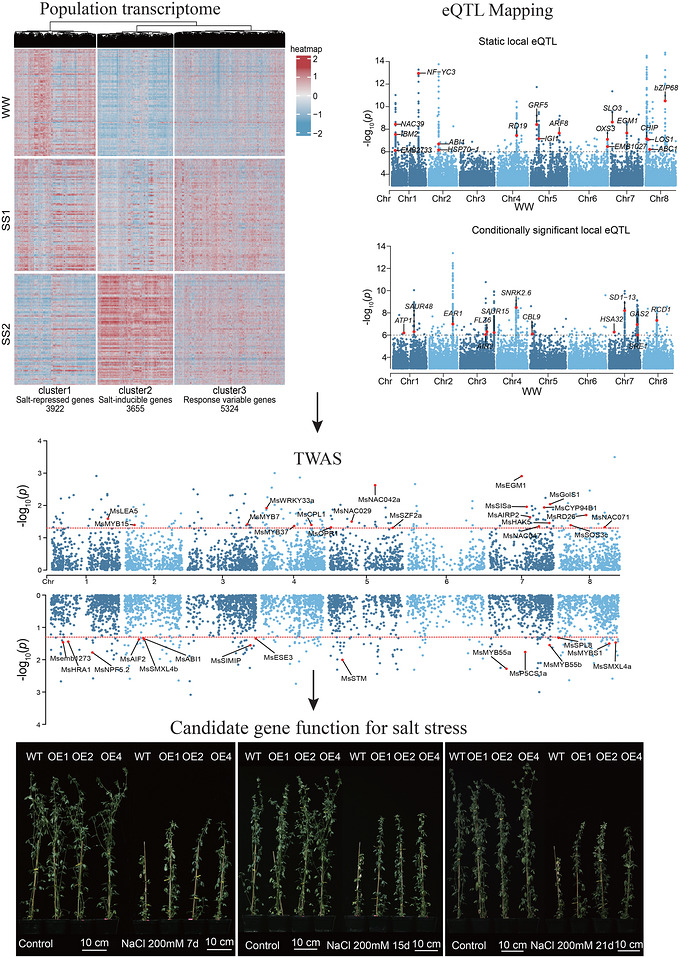

## 1 Introduction

Alfalfa (*Medicago sativa* L.) is among the most important forage crops worldwide, with a global planting area exceeding 32 million hectares that accounts for approximately 2.5% of the global agricultural crop planting area [1]. Owing to its high crude protein content and nutritional value and good palatability to livestock, alfalfa is known as the “king of forage” and is crucial to the dairy industry [2]. Salt stress is among the major environmental factors that limit global alfalfa production; therefore, mining the key genes involved in salt tolerance, exploring their natural functional variations and understanding their molecular regulatory mechanisms are critical for breeding salt‐tolerant varieties [3, 4].

Alfalfa is autotetraploid, and mining the regulatory genes of important agronomic traits using mutants is very difficult [5, 6]. Even if a mutant with extreme phenotypes is obtained, it is difficult to locate the mutant genes because of self‐incompatibility, making gene mining extremely difficult [5, 6]. On the basis of omics analyses (transcriptomics, proteomics and metabolomics) and homologous cloning from *Medicago truncatula*, which is a model species for alfalfa, several genes involved in salt tolerance in alfalfa, such as *MsSPL8*, *MsWRKY33*, *MsSPL12*, and *MsMYB206*, have been cloned [7, 8, 9, 10]. However, the regulatory network and favorable alleles of these genes are not fully understood. In recent years, genome‐wide association studies (GWASs) have been widely applied in crops such as alfalfa to elucidate natural variations associated with complex traits such as salt tolerance and flowering time [11, 12, 13, 14]. However, only a few genes have been identified by GWAS, which may be due to the complexity of the genetic basis of salt tolerance. Although GWASs can help detect associations between genetics and phenotypes, identification of causal genes or regulatory variants that mechanistically control complex traits is still challenging [15, 16, 17, 18].

Gene expression is the key to linking genotypes with phenotypes and is crucial to stress response and environmental adaptation [19]. Variations in gene expression, which are also known as expression quantitative trait loci (eQTLs), are regulated by *cis*‐ and/or *trans*‐regulation [20]. *Cis*‐eQTLs, also known as local‐eQTLs, tend to occur near target genes and alter expression levels owing to different variations in *cis*‐acting elements [21, 22]. In contrast, *trans*‐eQTLs, also known as distal‐eQTLs, tend to be located far from target genes, even on different chromosomes, and may indirectly affect the expression of target genes by modulating transcription factors or other complex signaling pathways [23, 24]. Further analysis of eQTLs in natural populations can reveal the genetic basis of natural and phenotypic variations at the expression level, which has been conducted in maize, rice, wheat and other plants [18, 25, 26, 27, 28, 29, 30, 31]. Because gene expression is closely related to different developmental stages and different tissues, the architecture of eQTLs can be conditionally significant during development or under environmental stimulation. In maize, researchers have performed eQTL analysis of population transcriptome data under different levels of drought stress and identified complex regulatory roles for eQTLs in modulating the molecular genetic basis of drought tolerance [18]. In rice, eQTL analysis using population transcriptome data under normal conditions and salt stress rapidly revealed the key rice salt tolerance gene STG5 [31]. In recent years, Mendelian randomization (MR) analysis and transcriptome‐wide association studies (TWASs) have been developed, which can identify candidate genes associated with certain traits through changes in gene expression and prioritize the inference of causal genes [18, 32, 33, 34]. Therefore, a comprehensive and accurate understanding of the plasticity of gene expression regulatory structures under salt stress is crucial, but related research in alfalfa is lacking.

Here, 528 RNA‐seq libraries from 176 alfalfa accessions were obtained under normal and salt stress conditions. By combining GWAS, population transcriptomics, TWAS, eQTL mapping and MR analysis, we identified several genetic loci and candidate genes significantly associated with salt tolerance in alfalfa. With a focus on conditionally significant regulatory variations, a putative regulatory network of transcription factors (TFs) involved in the response to salt stress was constructed. These findings offer insights into the complex basis of genetic regulation for salt tolerance and serve as a hypothesis‐generating resource for the breeding of salt‐tolerant alfalfa varieties.

## 2 Results

### 2.1 Genome‐Wide Association Studies Reveal the Complex Genetic Structure of Salt‐Related Traits

To identify candidate loci and genes conferring salt tolerance in alfalfa, an association panel of 176 germplasm accessions was used. Phenotypic evaluation was performed under normal and salt stress conditions, and the aboveground fresh weight (FW), dry weight (DW), and plant height (PH) were measured. Salt tolerance was assessed using the ratios of the phenotypic values under stress conditions to those under control conditions (FW_Salt/CK_, DW_Salt/CK_, and PH_Salt/CK_) and the salt tolerance index (STI). A GWAS with 4.76 million polymorphic markers, including 2.82 million SNPs and 2.05 million PAVs (Figure S1A, B) was conducted. Population structure analysis of the 176 germplasm accessions revealed three major subpopulations, which is consistent with previous findings (Figure S1C) [35].

**FIGURE 1:**
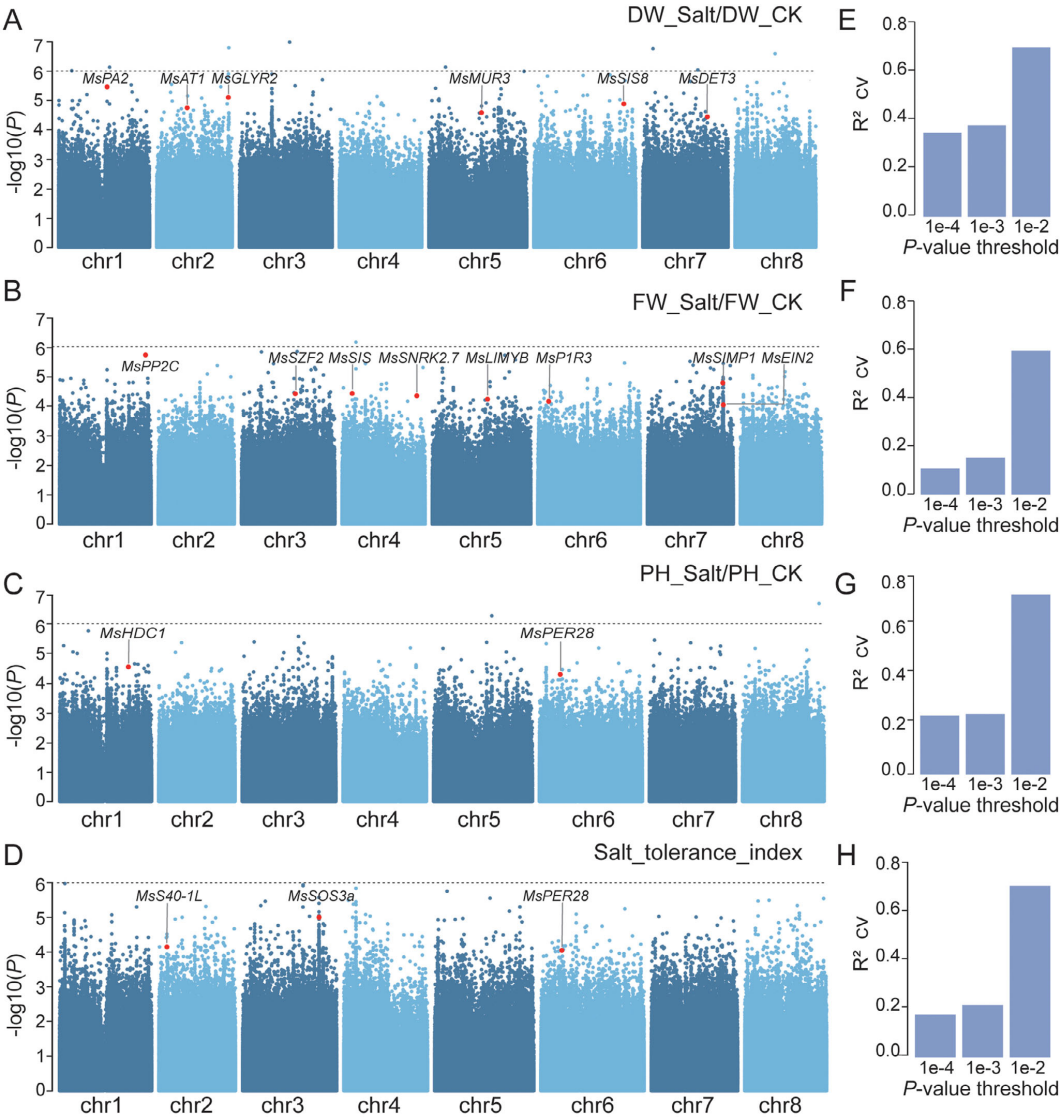
GWAS and genetic variant composition of salt‐related traits. (A—D), Manhattan plots of GWAS data using linear mixed models for DW_Salt/CK_, FW_Salt/CK_, PH_Salt/CK_ and the salt tolerance index. The black dashed lines indicate the threshold (*p* = 1.0 × 10^−6^), and the red points indicate the lead variants of significant sites. Genes that have been reported to be related to salt stress in plants are marked. E‐H, Phenotypic variance explained by the GWAS‐identified SNPs at different *p* value thresholds for DW_Salt/CK_, FW_Salt/CK_, PH_Salt/CK_ and the salt tolerance index. The explained phenotypic variances were assessed by tenfold cross‐validation using LASSO. *R^2^*, predicted values vs. observed phenotypes.

At a significance threshold of 10^−^^6^, multiple loci were significantly associated with the target traits. Under normal conditions, 8, 16, and 8 loci were associated with FW, DW, and PH, respectively. Under salt stress, these numbers were 7, 5, and 1, respectively. Among these, 1, 2, and 3 loci for FW, DW, and PH, respectively, were common to both conditions (Figure S2; Table S1). 8, 1, and 2 significant loci for DW_Salt/CK_, FW_Salt/CK_, and PH_Salt/CK_, respectively, were detected, whereas no significant locus was detected for STI (Figure 1A–D). Interestingly, some known salt‐responsive homologous genes, including *MsCBL4*, *MsCIPK3*, *MsPER28*, *MsPP2C*, and *MsSNRK2.7*, were detected below the significance threshold (Figure 1A–D) [36, 37, 38, 39, 40, 41]. Therefore, we hypothesize that salt tolerance in alfalfa is regulated by multiple minor‐effect genes or loci.

To test this hypothesis, the phenotypic variance explained (*R^2^* cv) by the GWAS loci at different thresholds was estimated using LASSO regression. The results revealed that at a threshold of 10^−^^4^, the value of *R^2^* cv was relatively low, generally less than 0.4 (Figure 1E–H). As the threshold was relaxed, the value of *R^2^* cv for these traits increased. At a threshold of 10^−^^2^, the *R^2^* cv values were 0.62, 0.73, 0.75, and 0.65 for the four traits, respectively (Figure 1E–H). These results indicate that the variation in salt stress tolerance in alfalfa may be primarily regulated by many minor‐effect loci.

### 2.2 Population Transcriptomic Analysis of Alfalfa in Response to Salt Stress

**FIGURE 2:**
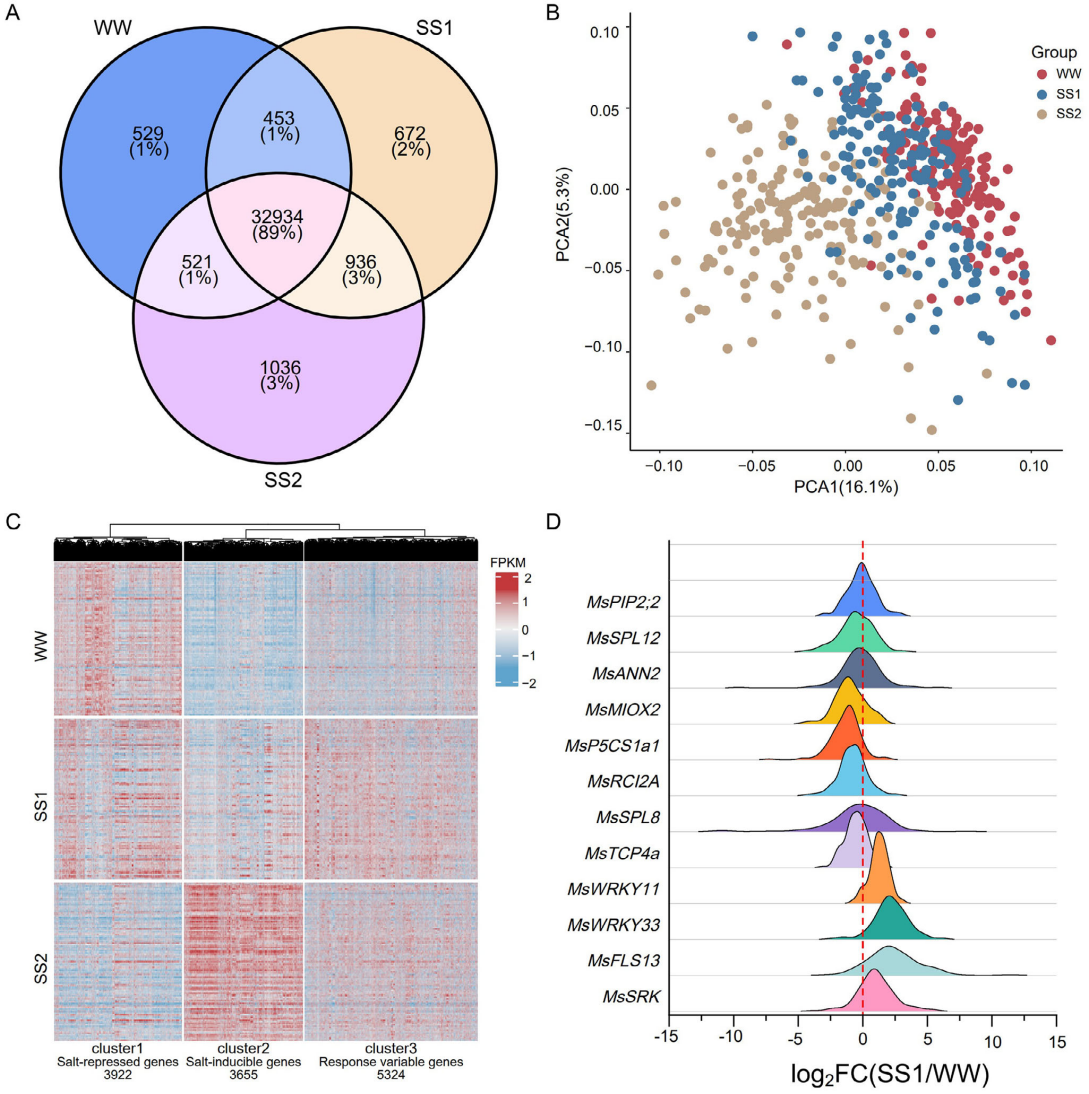
Population transcriptome analysis of 176 alfalfa accessions subjected to WW, SS1 and SS2 conditions. (A), Venn diagram illustrating the number of overlapping and uniquely expressed genes under WW, SS1 and SS2 conditions. (B), PCA separates the expression of identical genotypes under WW, SS1 and SS2 conditions. (C), Hierarchical clustering heatmap of 12,901 DEGs identified across all conditions. The color scale indicates normalized gene expression values, with red representing high expression and blue representing low expression. (D), Distribution of the log_2_ fold change in SS1/WW for known salt response‐related genes in alfalfa.

To identify genetic variants associated with transcriptional responses to salt stress, transcriptome analysis was conducted on the population under well‐watered (WW) and salt stress (SS) conditions at two time points: 2 days (SS1) and 5 days (SS2). Leaf samples from five plants per genotype under each treatment condition were pooled for bulk RNA sequencing. A total of 19.45 billion high‐quality reads were generated, with an average of 86.85 million reads per sample (Table S2). In total, 37,081 expressed genes were detected by mapping unique reads to the Zhongmu No. 1 reference genome, of which 32,934 genes were expressed across all conditions (Figure 2A). Principal component analysis (PCA) revealed clear separation among the WW, SS1, and SS2 groups, and transcriptomic differences increased with prolonged salt stress, indicating effective induction of gene expression (Figure 2B).

A total of 12,901 DEGs were identified on the basis of the criteria that they exhibit differential expression between any two treatments and be detected in more than 20% of the genotypes. The K‐means clustering algorithm was applied to classify these DEGs into three clusters. Cluster 1 (3,922 DEGs) and Cluster 2 (3,655 DEGs) represented salt‑repressed and salt‑induced genes, respectively, while the 5,324 DEGs in Cluster 3 displayed various expression patterns across these genotypes (Figure 2C). Heatmap visualization of these genes revealed that even the expression patterns of genes within the upregulated and downregulated clusters were not identical across genotypes. Numerous alfalfa genes associated with salt stress, including *MsWRKY11*, *MsWRKY33*, *MsFLS13* and *MsSRK*, were consistently activated across most accessions under SS1 conditions, whereas another set of genes, such as *MsSPL12*, *MsANN2, MsMIOX2*, *MsP5CS1a1*, *MsRCI2A*, *MsSPL8*, and *MsTCP4a*, was persistently suppressed (Figure 2D). Gene Ontology (GO) analysis revealed that the repressed DEGs were involved mainly in ethylene, leaf senescence, and phenylpropanoid biosynthetic processes (Figure S3A). The induced DEGs were involved in monoatomic ion homeostasis, cellular response to abiotic stimulus, and phloem or xylem histogenesis (Figure S3B). The variable DEGs were involved in plant‐type cell wall organization or biogenesis, carbohydrate biosynthetic processes, and the photosynthetic electron transport chain (Figure S3C). These results support the possibility that natural variation may lead to differences in gene regulation among different genetic backgrounds.

Given the importance of abscisic acid (ABA) in stress tolerance, the expression of ABA‑related genes was further examined. Among the 60 ABA‐associated DEGs, 15 genes (including *MsPYL1*, *MsPYL4*, *MsGPA1*, *MsCHC1* and *MsRAB28*) were uniformly downregulated, and 18 genes (including *MsNCED3*, *MsNAC002*, *MsMYB44*, *MsABF4* and *MsbZIP49*) were uniformly upregulated, while 27 genes (including *MsRD29B*, *MsMYB30*, and *MsABI8.2*) showed different responses across genotypes (Figure S4A–C). An integration of the GWAS results ( *p* < 1 × 10^−6^) revealed that 233 out of 731 GWAS candidate genes were also DEGs. Among these overlapping genes, 102 genes, such as *MsCBL4*, were consistently downregulated, 62 genes, such as *MsGLYR2* and *MsTCP10Ba*, were consistently upregulated, and 69 genes exhibited genotype‑specific expression patterns (Figure S5A,B; Table S3). These results indicate that natural variation mediates diverse transcriptional regulation and contributes to phenotypic differences in salt tolerance among alfalfa accessions.

### 2.3 Genome‐Wide Identification of Static and Conditionally Significant eQTLs for the Salt Stress Response

**FIGURE 3:**
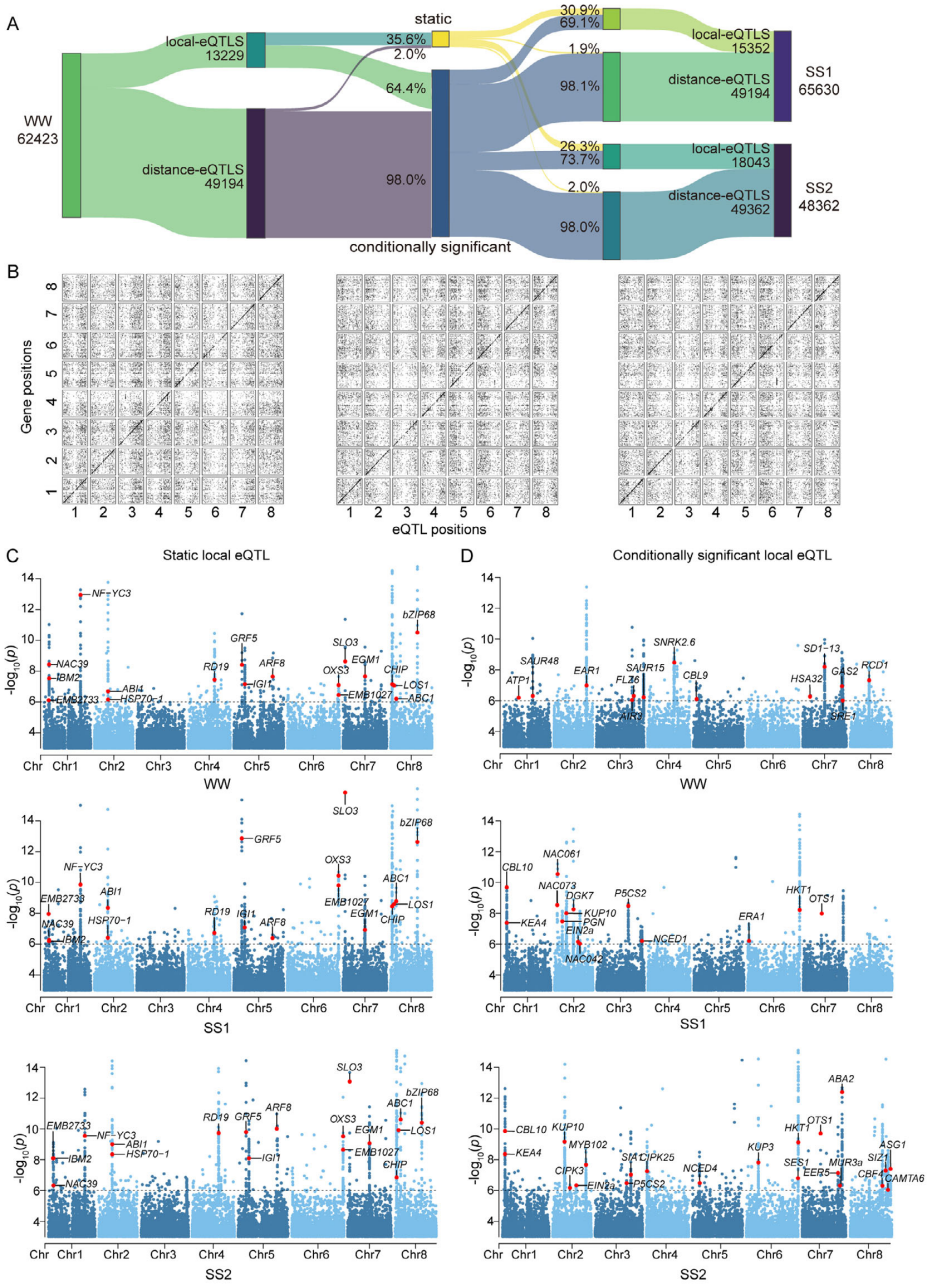
Large‐scale eQTLs identified by GWAS and conditionally significant changes in response to salt stress. (A), Sankey diagram showing the classification and proportional distribution of genome‐wide local‐ and distant‐eQTLs. The height of each node and the width of each flow are proportional to the number of eQTLs. Numbers in the nodes indicate the total count of eQTLs, and percentages on the flows indicate the proportion of eQTLs in each category relative to the source node. (B), Genome‐wide distribution of eQTL lead SNPs (x‐axis) and their associated eGenes (y‐axis) across the eight alfalfa chromosomes. The chromosomal positions of lead SNPs are plotted against the start positions of their target genes. Left: WW; Middle: SS1; Right: SS2. Points along the diagonal indicate local‐eQTLs (cis‐regulatory variants), while off‐diagonal points represent distant‐eQTLs (trans‐regulatory variants). (C), Combined Manhattan plots showing static local eQTLs — eGenes with significant local‐eQTL peaks consistently detected under all three conditions (WW, SS1, and SS2). The three rows correspond to WW, SS1, and SS2, respectively. Red points indicate lead SNPs, and labeled genes represent candidate regulators. (D), Combined Manhattan plots showing conditionally significant local eQTLs — eGenes exhibiting local‐eQTL peaks that vary in significance across conditions. The three rows correspond to WW, SS1, and SS2, respectively. For clarity, only locally significant eQTL peaks exceeding the significance threshold (p = 1.0 × 10^−6^, horizontal dashed line) are displayed for the target eGenes, along with randomly selected sub‐threshold marker loci.

To identify genetic regulatory variants associated with salt‐responsive gene expression in alfalfa, eQTL mapping was performed for all expressed genes. To avoid random errors, an eQTL was recognized only if at least three significant SNPs were detected under each condition. In total, 62,423, 65,630, and 67,405 eQTLs were identified under WW, SS1, and SS2 conditions, respectively. These eQTLs were associated with 11,606, 11,258, and 11,808 eGenes, representing 33.7%, 32.2%, and 33.3% of all expressed genes under the corresponding conditions, respectively (Figure 3A; Table S4). Among these eGenes, 278, 353, and 375 were regulated by ten or more eQTLs in WW, SS1, and SS2, respectively (Figure S6A).

Comparisons between eGene start positions and eQTL locations revealed strong diagonal enrichment under all conditions, indicating extensive local regulation of gene expression (Figure 3B). All eQTLs were classified as local (*cis*) and distant (*trans*) eQTLs by using a 1‐Mb upstream or downstream distance from the eQTL to its target eGene as the boundary. Under WW, SS1, and SS2 conditions, 21.2%, 23.4%, and 26.8% of the eQTLs were local, whereas 78.8%, 76.6%, and 73.2% were distant, respectively (Figure 3A). Further analysis revealed that 61.7% of the local eQTLs were located within 10 kb of the transcription start sites of their target eGenes, supporting the critical role of proximal regulatory elements (Figure S6B). In addition to the prevailing local regulation, the high proportion of distant eQTLs implied a complex regulatory network, and most eGenes regulated by local eQTLs were also controlled by distant eQTLs (Figure S7), highlighting the coordination between *cis* and *trans* regulation.

On the basis of the presence of eQTLs across treatments, the eQTLs detected in all three conditions were defined as static eQTLs, whereas those present in only one or two conditions were classified as conditionally significant eQTLs. In total, 4750 static local and 1000 static distant eQTLs were identified (Figure 3A). Moreover, 8,479, 10,602, and 13,293 conditionally significant local eQTLs as well as 48,194, 49,278, and 48,362 conditionally significant distant eQTLs were detected under WW, SS1, and SS2 conditions, respectively (Figure 3A). These results indicate that the majority of eQTLs are condition‐specific, with an increasing number of conditionally significant eQTLs detected under salt stress compared with normal conditions. Static local eQTLs accounted for 30.6% of all local eQTLs and were associated with 552 genes related to plant growth and development (e.g., *NF‐YC3*, *EMB2733*, *IBM2*, *GRF5*, *ARF8*, *IGI1*, *SLO3*, *EMB1027*, *NAC039*, *LOS1*, and *ABC1*), ABA signaling (e.g., *ABI1*, *RD19*, *bZIP68*, and *CHIP*), and reactive oxygen species pathways (e.g., *EGM1*, *OXS3*, and *HSP70‐1*) (Figure 3C). Conditionally significant local eQTLs and their corresponding eGenes were more abundant than static eQTLs under both normal and salt stress conditions (Figure S8A,B). The annotations of these target eGenes included ABA signaling (e.g., *EAR1*, *SD1‐13*, *GAS2*, *SRE1*, *EIN2*, *NCED1*, *ERA1*, *NCED4*, and *ABA2*), transcriptional regulation (e.g., *NAC073*, *NAC061*, *NAC042*, *CAMTA6*, *MYB102*, and *CBF4*), Ca^2+^ related (e.g., *CBL9*, *SOS3*, and *CIPK3*), K^+^ related (e.g., *KEA4*, *KUP3, KUP10*, and *HKT1*), and the salt stress response (e.g., *PGN*, *SES1*, and *SIZ1*) (Figure 3D). Among the 1,277 eGenes regulated by conditionally significant local eQTLs under WW, 65.9% were also detected under salt stress conditions (either SS1 or SS2), while 34.1% were specific to WW (Figure S8C). eGenes uniquely regulated by salt‐induced conditionally significant local eQTLs were enriched in the AP2, NAC, bHLH, and MYB families and functional terms related to cell wall biogenesis, ABA catabolism, and the osmotic stress response (Figure S9A,B). Collectively, these results suggest that both local and distant genetic variations contribute to stress‐responsive gene expression in alfalfa, with a substantial proportion of regulatory associations being condition‐specific.

### 2.4 Identification of eQTL Hotspots and Construction of a Predictive TF‐eGene Interaction Network

**FIGURE 4:**
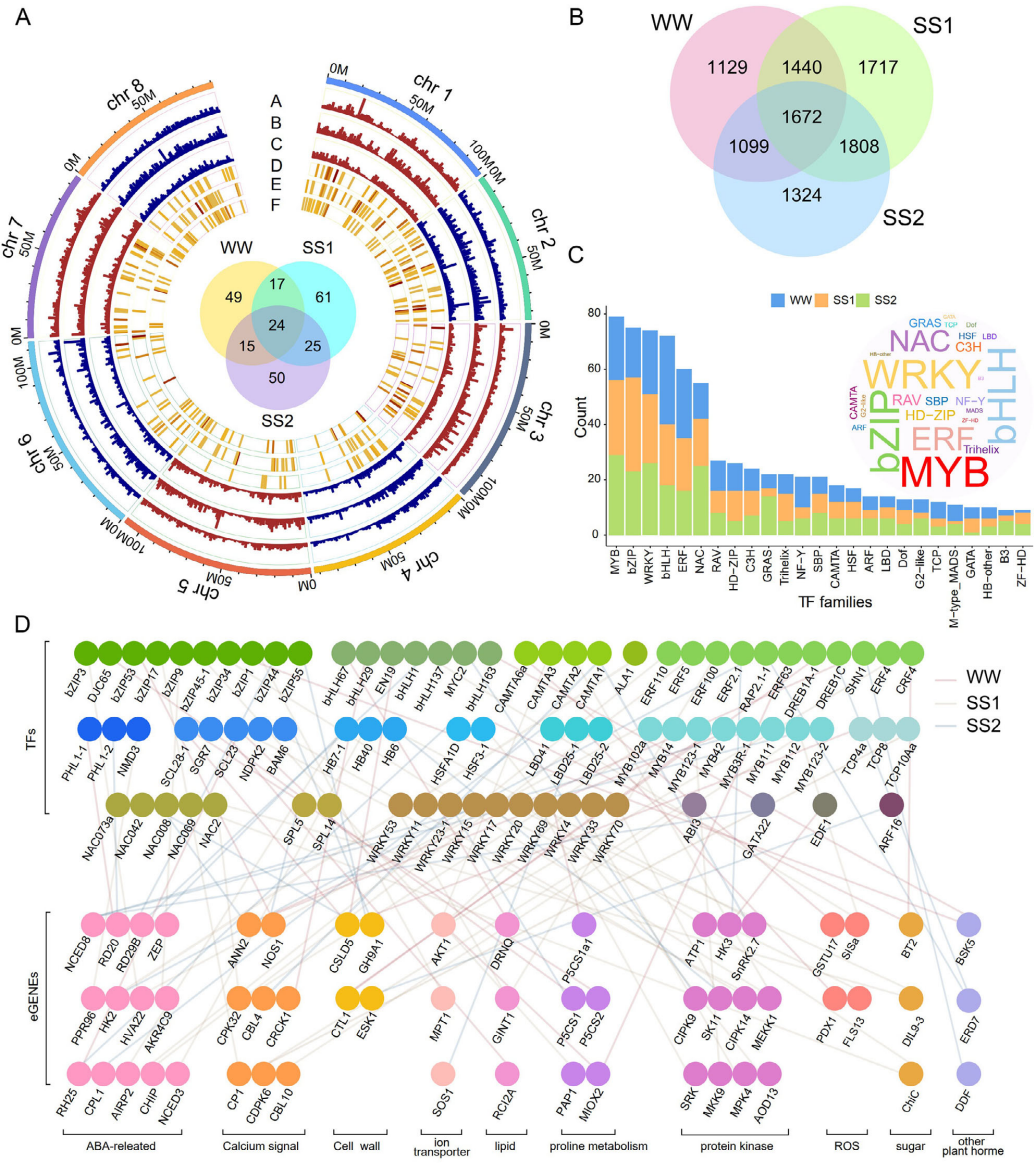
Identification of distance‐eQTL hotspots and construction of a transcription factor‐eGene regulatory network. (A), Circos plot showing the genomic distribution of distance‐eQTLs and distance‐eQTL hotspots across the eight chromosomes (chr1‐chr8) of alfalfa. Tracks A, B, and C (inner three rings) represent the distribution density of distance‐eQTLs under well‐watered (WW), mild salt stress (SS1), and severe salt stress (SS2) conditions, respectively. Tracks D, E, and F (outer three rings) display the genomic locations of distance‐eQTL hotspots, with red, yellow, and blue bars indicating hotspots under WW, SS1, and SS2, respectively. The inset Venn diagram shows the overlap of distance‐eQTL hotspots across the three conditions. (B), Venn diagram showing the number of eGenes whose expression is regulated by distant‐eQTL hotspots under WW, SS1, and SS2 conditions. (C), Bar chart (left) showing the number of transcription factor (TF) families enriched in distant‐eQTL hotspot regions across the three conditions, as identified by the XGBoost (eXtreme Gradient Boosting) regression model. The word cloud (right) displays the relative abundance of TF families, with font size proportional to frequency. (D), TF–eGene interaction network constructed based on upstream TFs predicted by the XGBoost regression model. The top layer shows TFs grouped by family (bZIP, bHLH, NAC, WRKY, etc.), colored by condition: green (WW), blue (SS1), and cyan (SS2). The middle layer represents eGENEs (target genes regulated by the TFs). The bottom layer categorizes eGENEs into functional groups (ABA‐related, calcium signal, cell wall, ion transporter, lipid, proline metabolism, protein kinase, ROS, sugar, and other plant hormones). Gray connecting lines indicate predicted TF‐eGene regulatory relationships.

Analysis of the genomic distribution of distant eQTLs revealed that 29.47% of the eQTLs were clustered in high‑density regions, and 346 chromosomal hotspots were identified (Figure 4A). Each hotspot was found to contain 98 to 358 distant eQTLs and to correspond to 22 to 107 targeted eGenes (Figure 4A; Table S5). These hotspot regions were estimated to account for approximately 30% of all distant eQTLs, while nearly 47% of eGenes were regulated by these regions. Under WW, SS1, and SS2 conditions, 105, 127, and 114 hotspots, respectively, were detected. These hotspots contained 26.7%, 32.1%, and 29.4% of the distant eQTLs identified under each condition, and 45.1%, 49.3%, and 45.5% of the corresponding eGenes were regulated by these hotspots, respectively. Comparisons of eQTL hotspots between normal and salt stress conditions revealed that 56 hotspots (23.2%) were shared, whereas 185 hotspots (76.7%) were specific to either normal (49) or salt stress conditions (136). The number of unique hotspots induced by salt stress was nearly three times greater than that under normal conditions (Figure 4A). It was also found that 4,211 eGenes (41.3%) were regulated by shared hotspots, whereas 5,978 eGenes (58.7%) were regulated by condition‑specific hotspots (Figure 4B). These results indicated that more unique distant eQTL hotspots were induced by salt stress than the stably shared subset, reflecting a strong and distinct transcriptional regulatory response.

Given the well‐characterized mechanisms of plant salt stress responses, 57 eGenes with literature‐curated stress‐related functional annotations were selected for further analysis (Table S6). These eGenes are involved in ABA biosynthesis and signaling, Ca^2+^ signaling, reactive oxygen species scavenging, ion transport, cell wall formation, and other plant hormone and protein kinase pathways. To identify potential transcriptional regulators, we applied an XGBoost regression model based on gradient boosting decision trees to capture statistical associations between transcription factors (TFs) within eQTL hotspots and the expression of these 57 stress‐related eGenes. In total, 240 TFs from more than 25 families, mainly MYB, bZIP, WRKY, bHLH, ERF, and NAC, were identified (Figure 4C). On the basis of the significant statistical associations between 81 TFs and 57 eGenes, we constructed a putative TF‐eGene regulatory network (Figure 4D), which serves as a comprehensive, hypothesis‐generating framework for understanding the molecular mechanisms of salt stress adaptation in alfalfa. These predicted interactions require experimental validation.

### 2.5 Twas Identifies Candidate Salt Tolerance Genes Regulated by Many Small‐Effect Loci

**FIGURE 5:**
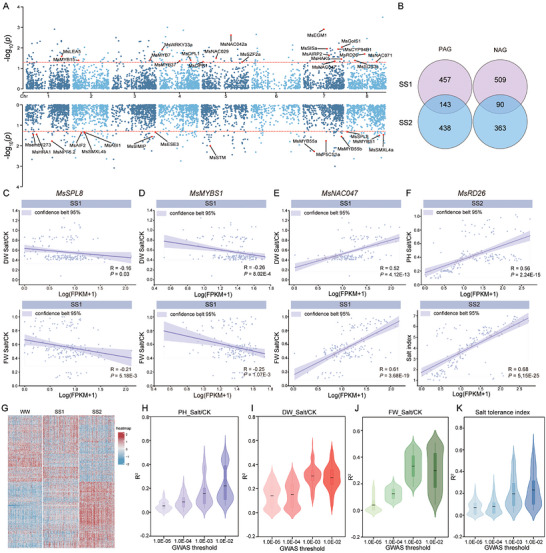
Transcriptome‐wide association study (TWAS) for salt‐related traits. (A), Manhattan plot of the TWAS for DW_Salt/CK_, FW_Salt/CK_, PH_Salt/CK_ and the salt tolerance index under SS1 and SS2 conditions. The red dashed lines indicate *p* values of 0.05. The genes reported to be related to abiotic stress in alfalfa and other plants are marked. (B), Venn diagram illustrating the number of overlapping and unique PAGs and NAGs identified under SS1 and SS2 conditions. (C–F), Correlation analysis between the expression of MsSPL8, MsMYBS1, MsNAC047, and MsRD26 under SS1 or SS2 conditions and the traits for DW_Salt/CK_, FW_Salt/CK_, PH_Salt/CK_ and the salt tolerance index. G, Expression patterns of the 300 most significant TWAS genes associated with DW_Salt/CK_, FW_Salt/CK_, PH_Salt/CK_ and the salt tolerance index in the 176 varieties under WW, SS1 and SS2 conditions. Each column represents a gene, and each row represents a variety. H‐K, *R^2^* the square of the Pearson correlation coefficient between the four traits and the predicted expression values of the top 300 TWAS significant genes. The expression values were predicted using the LASSO model based on lead variants of GWAS QTLs identified at different thresholds in the 176 accessions.

To investigate associations between gene expression and salt‐related traits, TWAS was performed. At a false discovery rate (FDR) threshold of 0.05, 1,758, and 1,681 significant genes were identified under SS1 and SS2, respectively, for multiple salt tolerance traits (Figure 5A, Figure S10, Table S7). In total, 1,700 negatively associated genes (NAGs) and 1,739 positively associated genes (PAGs) were detected, with 218 NAGs and 254 PAGs shared at both time points (Figure S11A). With respect to DW_Salt/CK_, FW_Salt/CK_, PH_Salt/CK_ and STI, 962 NAGs and 1,038 PAGs were detected, including known salt‐responsive genes such as *MsMYB15*, *MsSISa*, *MsSPL8*, and *MsSOS3* (the alfalfa ortholog of Arabidopsis AtSOS3) (Figure 5A). Among them, 90 NAGs and 143 PAGs were stably detected under both SS1 and SS2 conditions (Figure 5B). NAGs were enriched mainly in the MYB, bZIP, JAG, and NF‐YC TF families, whereas PAGs were enriched in the WRKY and NAC families (Figure S11B).

Among these genes, some genes related to salt stress in alfalfa or homologs of Arabidopsis were found. *MsSPL8* encodes an SBP TF and has been reported to be a negative regulator of salt tolerance in alfalfa [7]. *MsSPL8* expression was negatively correlated with DW_Salt/CK_ (*R* = ‐0.16; *p* = 0.03) and FW_Salt/CK_ (*R* = ‐0.21; *p* = 5.2 × 10^−3^) (Figure 5C). *MsMYBS1* encodes a MYB TF and its homolog, *AtMYBS1*, has been reported to be involved in the salt response in Arabidopsis [42]. *MsMYBS1* expression was negatively correlated with DW_Salt/CK_ (*R* = ‐0.26; *p* = 8.0×10^−4^) and FW_Salt/CK_ (*R* = ‐0.25; *p* = 1.1×10^−3^) (Figure 5D). Among the PAGs, the expression of *MsNAC047*, which encodes an NAC TF, was positively correlated with DW_Salt/CK_ (*R* = 0.52, *p* = 4.1×10^−13^) and FW_Salt/CK_ (*R* = 0.61, *p* = 3.7×10^−19^) (Figure 5E). The expression of another NAC TF, MsRD26, was positively correlated with PH_Salt/CK_ (*R* = 0.56, *p* = 2.2×10^−15^) and the salt tolerance index (*R* = 0.68, *p* = 5.2×10^−25^) (Figure 5F). In addition to these TFs, other functional genes, including the galactinol synthase gene *MsGolS1*, the oxylipin metabolism‑related gene *MsOPR1*, and the high‐affinity K^+^ transporter gene *MsHAK5*, were significantly positively correlated with salt stress in alfalfa (Figure S12A–C). Previous studies have reported that homologous genes in Arabidopsis and other plants can increase salt tolerance or affect the salt response [43, 44, 45, 46, 47, 48, 49]. In summary, the TWAS results demonstrate that our data are valuable for revealing the genes and regulatory networks related to salt stress.

To explore the expression patterns of the genes associated with salt stress identified by TWAS, we found that many genes displayed similar expression profiles under salt stress (Figure 5G). Considering that PH_Salt/CK_, DW_Salt/CK_, FW_Salt/CK_ and STI are regulated mainly by small‐effect genetic loci, we speculate that the variations in the expression of significant TWAS genes for these traits may also be regulated primarily by small‐effect loci. To verify this hypothesis, LASSO regression was applied to predict the expression of the 300 most significant TWAS genes associated with each trait using the GWAS lead variants at multiple thresholds. The Pearson correlation coefficients (R^2^ values) between the predicted gene expression and the corresponding salt stress‐related traits were low at the threshold of 10^−^^4^ but were significantly greater at the more relaxed thresholds of 10^−^^3^ and 10^−^^2^ (Figure 5H–K). Thus, our results suggest that expression of these TWAS genes are largely associated with minor‐effect loci.

### 2.6 Integration of TWAS and Mendelian Randomization Prioritizes Candidate Salt Tolerance Genes

**FIGURE 6:**
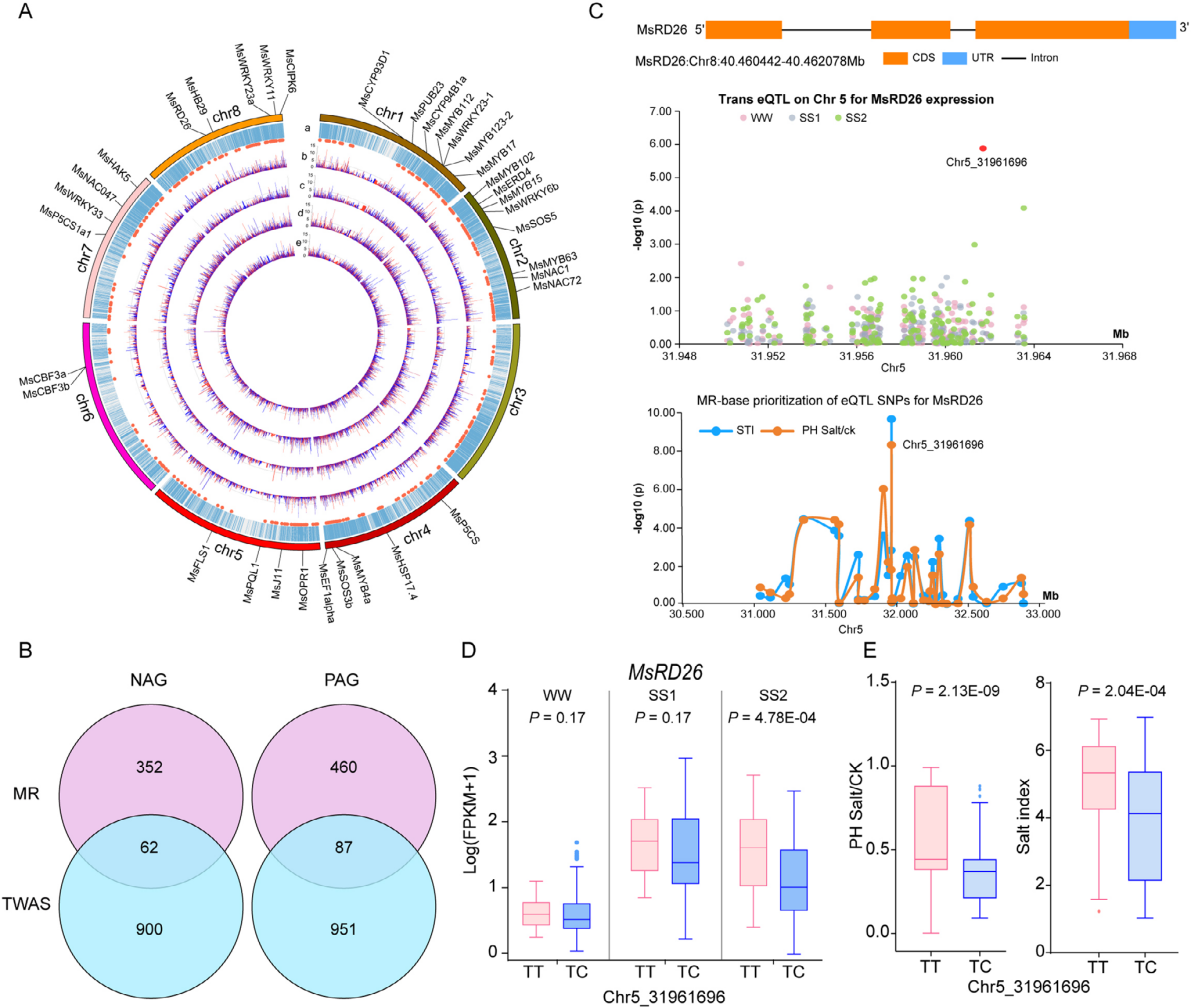
MR tests of the associations between gene expression and the salt stress‐related phenotype. (A), Genes identified as related to these salt stress‐related traits. Tracks b, c, d, and e represent the MR test results for DW_Salt/CK_, FW_Salt/CK_, PH_Salt/CK_ and the salt tolerance index. The y‐axis is the ‐log_10_ (*p* value) value of the MR test significance. The gray lines indicate the threshold of 1 × 10^−5^. Each line represents an individual gene. The red and blue lines indicate that the expression of this gene was positively or negatively correlated with the four traits, respectively. Track a represents genes whose expression was associated with at least two unique traits. Each orange point represents an individual gene. Some genes or their homologous genes related to abiotic stress in alfalfa or other plants are marked. (B), Venn diagram illustrating the number of overlapping and unique PAGs and NAGs identified by the TWAS or MR test. (C), Genomic characterization of MsRD26 and its trans‐eQTL signals. The top panel shows the gene structure of MsRD26 at its exact genomic position on Chr 8 (40.460442‐40.462078 Mb). The middle panel presents the Manhattan plot of trans‐eQTLs for MsRD26 expression located on Chr 5 (31.9 Mb region), with the lead SNP (Chr5_31961696, −log_10_(P) = 5.88) highlighted in red. The bottom panel shows the MR‐based prioritization of eQTL SNPs for MsRD26, displaying the SNP associations with STI (blue, −log_10_(P) = 9.64) and PH_Salt/CK_(orange, −log_10_(P) = 8.29). (D), Allelic comparison of MsRD26 gene expression levels under WW, SS1 and SS2 conditions based on the SNP (Chr5_31961696). E, Allelic comparison of PHSalt/CK and the salt tolerance index based on the SNP (Chr5_31961696). The sample sizes for the TT and TC genotype groups are n = 31 and n = 121, respectively. Statistical significance was determined by two‐sided Student's t‐test. P values are shown in the figure.

To identify genes whose expression levels (with identified regulatory variants) are relevant to plant salt tolerance, a Mendelian randomization (MR) test was conducted. At the threshold of *p* < 1 × 10^−5^, 1,401 candidate genes were prioritized for their contribution to salt‐related traits, including DW_Salt/CK_, FW_Salt/CK_, PH_Salt/CK_, STI and the absolute values of DW, FW, and PH under salt stress, on the basis of their alterations in expression levels (Figure 6A, Figure S13, Table S8). Overall, 650 genes were found to negatively contribute to these salt‐related traits, and 751 genes were positively associated with these traits. Afterward, we focused on genes significantly associated with the four traits of DW_Salt/CK_, FW_Salt/CK_, PH_Salt/CK_ and STI. A total of 414 NAGs and 547 PAGs, including MYB TFs (*MsMYB102*, *MsMYB112*, *MsMYB12*, *MsMYB15*, *MsMYB17*, *MsMYB4*, and *MsMYB63*), NAC TFs (*MsNAC1*, *MsRD26*, and *MsNAC047*), and WRKY TFs (*MsWRKY11*, *MsWRKY23a*, *MsWRKY23b*, *MsWRKY33a*, and *MsWRKY6b*), were significantly associated with these four plant traits (Figure 6A). Among these genes, 62 NAGs and 87 PAGs, such as *MsRD26*, *MsOPR1*, *MsHAK5*, *MsNAC047* and *MsMYB15*, were identified by both TWAS and MR analysis (Figure 6B).

Further mechanistic analysis of candidate genes revealed their distinct regulatory architectures. The expression of the NAC transcription factor *MsRD26*, a putative positive regulator, is regulated by a conditionally significant distant eQTL (Figure 6C), with the TT allele linked to higher expression under SS2 conditions and improved salt tolerance (Figure 6D, E). Similarly, for another NAC transcription factor, *MsNAC047*, of which the AA allele of its eQTL is a superior allele, as accessions carrying it exhibit both increased expression under SS1 conditions and enhanced salt tolerance (Figure S14A). The MYB transcription factor *MsMYB15* is also regulated by a specific conditionally significant distant eQTL, where the TC allele results in its increased expression under stress and greater salt tolerance (Figure S14B). In addition, the expression of *MsOPR1* (encoding a 12‐oxo‐phytodienoic acid reductase) is governed by an eQTL for which the AG allele is associated with upregulated expression under SS2 and superior salt tolerance (Figure S14C). All prioritized genes represent candidates for future functional validation and allelic mining.

### 2.7 Overexpression of MsRD26 Significantly Increases Salt Tolerance in Alfalfa

**FIGURE 7:**
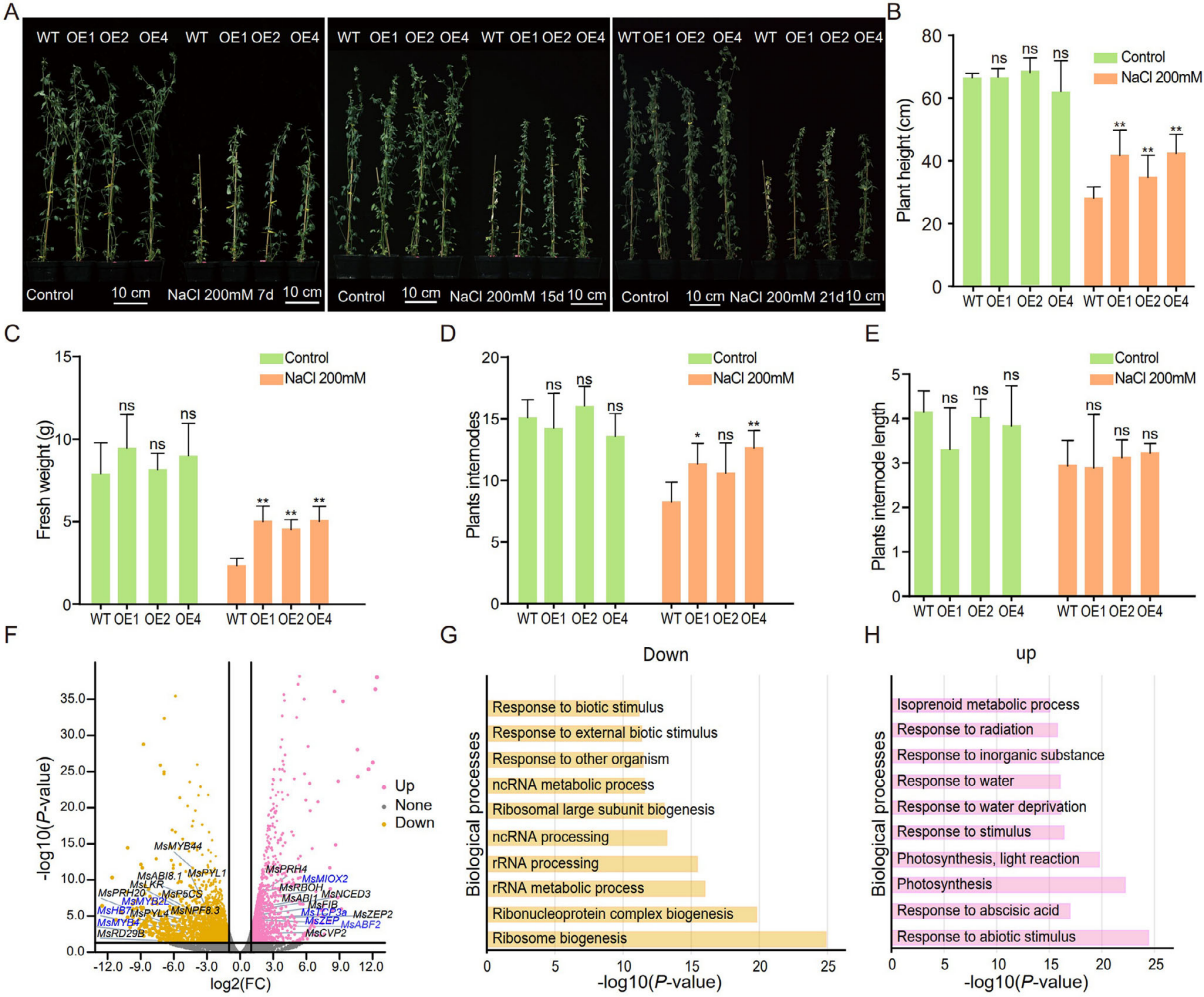
*MsRD26* positively regulates salt tolerance in alfalfa. (A), Phenotypic comparison of wild‐type (WT) and MsRD26‐overexpression (OE1, OE2, OE4) plants under normal (Control) and salt stress (200 mM NaCl) conditions for 7, 15, and 21 days (from left to right). Scale bar = 10 cm. (B–E), Quantitative analysis of plant height B, fresh weight C, internode number D, and internode length E, in WT and OE lines under normal and salt stress (200 mM NaCl, 21 days) conditions. Data are presented as means ± SE (n = 3 biological replicates per group). Statistical significance was determined by Student's t‐test. ns indicates no significant difference, * indicates significant difference at p < 0.05, ** indicates significant difference at p < 0.01. (F), Volcano plot showing differentially expressed genes (DEGs) between WT and MsRD26‐OE4 under salt stress (200 mM NaCl, 21 days). Yellow and magenta dots represent downregulated and upregulated genes, respectively; gray dots indicate non‐significant genes. Blue‐labeled gene names are associated with salt stress responses; black‐labeled gene names are associated with the ABA signaling pathway. DEGs were defined as genes with |log_2_FC| > 1 and adjusted *p*‐value (FDR) < 0.05. (G,H), GO analysis of the DEGs between the WT and MsRD26‐OE4 lines under salt stress. G indicates downregulated genes, and H indicates upregulated genes.

To validate whether the genes identified above are associated with salt stress tolerance, three independent transgenic MsRD26‐overexpressing (MsRD26‐OE) lines were generated (Figure S15). Under normal growth conditions, there were no significant differences in plant height, fresh weight, or overall growth between the wild‐type (WT) and MsRD26‐OE transgenic alfalfa lines. However, after 7 days of salt stress, the leaves of the WT lines began to wilt and become dehydrated (Figure 7A). With prolonged stress, their leaves gradually turned yellow and eventually died (Figure 7A). In contrast, the growth of the transgenic lines remained normal throughout the salt stress period. After 21 days of treatment, the MsRD26‐OE transgenic lines exhibited significant increases in both plant height and fresh weight, which exceeded those of the wild type by 50%–55% (Figure 7B, C). Further analysis revealed that the increase in plant height of the MsRD26‐OE transgenic lines was primarily due to a greater number of nodes rather than longer internodes (Figure 7D, E; Figure S16A). Physiological assays revealed that under salt stress, compared with the WT plants, the MsRD26‐OE transgenic lines presented significantly greater superoxide dismutase (SOD) and peroxidase (POD) activity (Figure S16B, C). Taken together, these results demonstrated that overexpression of *MsRD26* could improve salt tolerance in alfalfa, potentially by increasing the activity of the antioxidant enzyme system.

To better understand the molecular mechanism of *MsRD26* under salt stress, RNA‐seq was conducted on the salt‐stressed MsRD26‐OE4 and WT lines. The analysis revealed that under salt stress, compared with those in the WT line, the expression levels of 4,158 genes significantly changed in the OE4 line, including 2,423 genes that were upregulated and 1,735 genes that were downregulated (Figure 7F, Table S9). These differentially expressed genes (DEGs) included several genes in alfalfa that are known to respond to salt stress, such as *MsMIOX2*, *MsMYB2L*, *MsTCP3a*, *MsZFP*, and *MsMYB4*, and multiple ABA pathway‐related genes, such as *MsABF2*, *MsPYL1*, *MsNCED3*, and *MsABI8.1*. GO analysis revealed that these upregulated genes were enriched mainly in biological pathways such as response to abiotic stimulus, response to abscisic acid, and response to water deprivation, whereas the downregulated genes were enriched mainly in pathways such as ribosome biogenesis, ribonucleoprotein complex biogenesis, and rRNA metabolic process (Figure 7G, H). These results indicate that *MsRD26* may regulate salt tolerance in alfalfa through multiple pathways.

### 2.8 Genetic Basis of Alfalfa Salt Tolerance and Genome‐Wide Predictions

**FIGURE 8:**
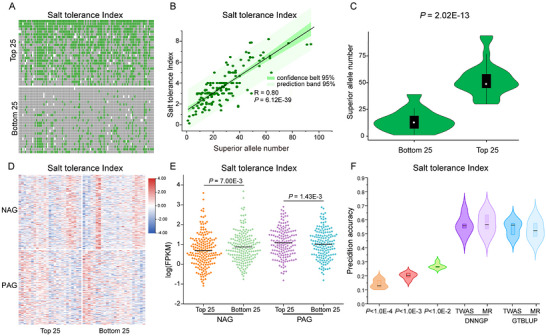
Genetic basis of the development of salt tolerance and genome‐wide predictions. (A), Distribution of superior alleles between the top 25 salt‐tolerant materials (Top25) and the bottom 25 salt‐tolerant materials (Bottom25). Among them, green boxes represent superior alleles, gray boxes represent unfavorable alleles, and white boxes represent missing alleles. The significance of allele frequency differences was assessed by Fisher's exact test with FDR correction for multiple testing (Table S12). (B), Correlations between the number of superior alleles and the salt tolerance index. (C), Comparison of the number of superior alleles between Top25 and Bottom25. n = 25 accessions per group. Statistical significance was determined by two‐sided Student's t‐test. P values are shown in the figure. (D), Heatmap showing the expression patterns of NAG and PAG genes associated with the salt tolerance index identified by the TWAS. Each column represents a gene, and each row represents a variety. (E), Comparison of the expression of NAGs and PAGs among Top25 and Bottom25 under the SS2 condition. Beeswarm plots showing the distribution of average expression values of NAGs (185 genes) and PAGs (159 genes) in Top25 and Bottom25 accessions under SS2 conditions. Each point represents an individual gene. For each accession, the mean expression level of all NAGs (185 genes) or PAGs (159 genes) was calculated. Statistical significance between Top25 and Bottom25 groups was assessed by two‐sided Student's t‐test. P values are shown in the figure. (F), Genome‐wide prediction of the salt tolerance index on the basis of significant SNPs at different thresholds and the expression of genes identified by the TWAS or MR test. Genome‐wide predictions of the SNPs were conducted by the GTBLUP model. Genome‐wide predictions of gene expression were conducted by the DNNGP model.

To further reveal the genetic basis of salt tolerance in alfalfa, integrative analyses at the DNA and transcriptome levels were conducted. Haplotype analysis demonstrated that superior haplotypes associated with STI were significantly enriched in salt‐tolerant accessions (top 25%), with much greater frequencies than in salt‐intolerant accessions (bottom 25%) (Figure 8A, C). Correlation analysis revealed a strong positive correlation between superior haplotype abundance and STI (Figure 8B), and similar patterns were observed for DW_Salt/CK_, FW_Salt/CK_, and PH_Salt/CK_ (Figure S17). Fisher's exact tests confirmed that superior alleles were significantly enriched in the top 25% group compared to the bottom 25% group. For the STI trait, 89 out of 110 loci showed significant enrichment after FDR correction (adjusted p < 0.05). Similar enrichment patterns were observed for FW_Salt/CK_, DW_Salt/CK_, and PH_Salt/CK_ (Supplementary Table S12). At the expression level, TWAS‐identified PAGs were significantly upregulated in tolerant accessions, whereas NAGs were more highly expressed in intolerant accessions for STI and the three related traits (Figure 8D, E; Figure S18).

Finally, to evaluate the breeding application value of these findings, different models were applied to make genome‐wide predictions for these traits. The accuracy of the GTBLUP model improved with relaxed GWAS thresholds but remained below 0.4. In contrast, the DNNGP model, which incorporated gene expression data from the TWAS and MR analyses, exhibited greatly increased prediction precision (Figure 8F; Figure S19). These findings confirm that superior haplotypes and transcriptional regulation jointly underpin alfalfa salt tolerance and that gene expression provides a more effective predictor for molecular breeding.

## 3 Discussion

Salt tolerance in alfalfa is controlled by complex polygenic interactions, and determining its genetic mechanisms remains challenging for breeding [50]. Here, we integrated whole‐genome variations with population transcriptome data, employing GWAS, eQTL mapping, and TWAS to characterize natural variation in alfalfa salt tolerance and functionally validated a candidate regulator, *MsRD26*. Incorporating gene expression data into genomic prediction models substantially improved prediction accuracy compared to SNP‐based approaches.

GWAS serves as a powerful tool for elucidating the genetic architecture of complex traits [51]., but stringent multiple testing corrections often lead to the omission of genuinely associated loci [52]. Salt tolerance is typically governed by numerous small‐effect genes that fail to reach conventional significance under limited sample sizes [53]. For instance, studies on rice panicle development have demonstrated that overly strict thresholds can obscure established causal genes [54]. Similarly, our analysis revealed that applying a stringent threshold of *p* < 10^−^^6^ yielded only a handful of significant loci, while several known salt tolerance genes, including *MsCBL4* and *MsCIPK3*, were not detected. Progressive relaxation of the threshold led to a gradual increase in explained phenotypic variance, confirming that alfalfa salt tolerance is likely controlled by numerous minor‐effect loci [55].

eQTL mapping systematically identifies genetic variants that modulate gene expression [26, 56, 57]. and offers advantages over conventional GWAS for identifying stress‐responsive genes [31, 58, 59, 60, 61]. Here, we applied eQTL analysis to 528 RNA‐seq samples from 176 alfalfa accessions and identified 12,901 DEGs. Among these genes, 5,324 (41.2%) exhibited genotype‐dependent variable response patterns, a proportion substantially higher than that reported for wheat (18.1%) and maize (27.7%) under drought stress [18, 58], suggesting that alfalfa stress responses may be subject to more complex genetic background effects. This pattern was particularly evident in the ABA pathway, in which 27 of 60 differentially expressed genes (45.0%), including *MsZEP2* and *MsPYL7*, exhibited genotype‐specific responses, further underscoring the influence of genetic background on salt stress adaptation strategies. We identified 62,423, 65,630, and 67,405 eQTLs under WW, SS1, and SS2 conditions, corresponding to 11,606, 11,258, and 11,808 eGenes, respectively. While distant eQTLs predominated across all conditions, the proportion of local eQTLs increased progressively from 21.2% (WW) to 26.8% (SS2), indicating increased involvement of *cis*‐regulatory elements during salt stress adaptation. These findings contrast with studies on the response of wheat to drought, which showed stable local eQTL proportions [58], suggesting that alfalfa salt stress responses may involve a greater proportion of conditionally detected genetic associations, under the experimental conditions tested here. Further analysis revealed that 14,250 local eQTLs (30.6%) were consistently detected across all three conditions, a proportion substantially exceeding that of static distant eQTLs (2.0%), indicating high conservation of proximal regulatory variations. Furthermore, conditionally significant eQTLs constituted major fractions in both categories, revealing ubiquitous environment‐specific regulatory elements. Remarkably, more than 96% of the eGenes regulated by local eQTLs were also regulated by distant eQTLs, suggesting that this regulatory architecture may amplify local variant effects through transcription factor‐mediated *trans*‐acting elements. We caution that our classification of conditionally significant eQTLs is based on significance thresholds and does not formally test differential regulation across conditions. Furthermore, with 176 accessions, detection of very small‐effect eQTLs is limited, and our catalog is enriched for moderate‐to‐large effect variants. We also acknowledge that our eQTL mapping used a simplified diploid approach without explicit tetraploid dosage modeling.

Phenotypic metrics also warrant careful consideration. STI is a composite trait integrating multiple physiological responses rather than measuring a single adaptive process. Accordingly, GWAS and MR signals derived from STI should be interpreted as associations with overall visible salt tolerance rather than specific mechanisms. Nevertheless, the detection of known salt‐responsive genes such as MsCBL4 and MsPER28 near the significance threshold, and the functional validation of MsRD26, suggest that STI‐based GWAS captured genuine genetic signals. Our RNA‐seq design pooled five individuals per accession into a single library per condition, which provides stable genotype‐level expression estimates consistent with previous studies [62, 63], but sacrifices within‐genotype variation and does not allow assessment of sequencing technical noise.

Distant eQTL hotspots, which exhibit highly clustered genomic distributions and coordinate the expression of multiple downstream targets, have been documented in rice, maize, and wheat [18, 54, 64]. Our analysis revealed 346 such hotspots comprising merely 30% of the distant eQTLs yet regulating 47% of the eGenes, underscoring their important regulatory roles. Salt stress induced 136 specific hotspots, approximately threefold more than the number observed under normal conditions, and these hotspots regulated substantially greater proportions of eGenes. Consistent with previous findings of stress‐induced transcriptional network reprogramming [58], the higher proportion of stress‐specific hotspots in alfalfa may reflect the enhanced transcriptional regulatory plasticity that evolved in this perennial species to cope with persistent stress. The XGBoost‐based TF–eGene interaction network, which was successfully applied in wheat, maize, and rapeseed [18, 58, 64, 65, 66, 67], was extended here to alfalfa, revealing a regulatory backbone centered on the MYB, bZIP, and NAC families and providing resources for dissecting the stress response regulatory network. We emphasize that these predicted interactions are statistical associations and require experimental validation.

TWAS leverages gene expression data to infer functional associations between genes and phenotypes [68], complementing GWAS. Many TWAS‐significant genes exhibited differential expression controlled by multiple small‐effect loci, corroborating the polygenic inheritance pattern observed in GWAS [54]. Methods capable of capturing cumulative minor effects offer clear advantages over single‐locus GWAS strategies [69]. Among these candidate genes, *MsNAC047*, *MsRD26* and *MsMYB15*, which have homologs in Arabidopsis and wheat, have been confirmed to participate in the abiotic stress response [43, 44, 46, 48]. The overexpression of *MsRD26* significantly increased salt tolerance in alfalfa, which is consistent with the central role of NAC transcription factors documented for rice OsNAC5/6 and wheat TaNAC29 [46, 70, 71, 72]. Comparative transcriptome analysis of MsRD26‐OE transgenic lines and the wild type under salt stress revealed that *MsRD26* modulates the expression of multiple ABA‐related genes, including *MsABF2*, *MsPYL1*, and *MsNCED3* (Figure 7F–H). EMSA and dual‐luciferase assays confirmed that MsRD26 directly binds to the MsABF2 promoter and activates its transcription (Figure S20), suggesting that MsRD26 contributes to salt tolerance at least in part through ABA signaling. We acknowledge the methodological limitations of MR in plants, including the use of single‐SNP instruments. Our results are therefore interpreted as a prioritization tool for candidate nomination rather than definitive causal inference, with functional validation providing the critical test. Furthermore, we recognize that the current evidence for the functional role of *MsRD26* is primarily derived from gain‐of‐function (overexpression) experiments. While these results demonstrate that *MsRD26* is sufficient to enhance salt tolerance, they do not establish its necessity as a canonical positive regulator. Complementary loss‐of‐function analyses (e.g., CRISPR/Cas9‐mediated knockout or RNA interference) will be required to rigorously test the requirement of *MsRD26* in the salt stress response. Accordingly, we refer to *MsRD26* as a putative positive regulator of salt tolerance throughout this study, rather than a conclusively defined one.

Beyond these findings, this study revealed the genetic basis of salt tolerance in alfalfa on the basis of DNA haplotypes and gene expression. Superior haplotypes were significantly enriched in salt‐tolerant materials and positively correlated with the salt tolerance index, which is consistent with the conclusions of many GWAS‐based studies on excellent haplotype screening of crops [73, 74]. Moreover, the prediction accuracy of the DNNGP model, which included gene expression data, was significantly greater than that of the SNP‐based GTBLUP model alone. These findings align with the recent trend of using transcriptome data to improve the prediction accuracy of complex traits reported in other studies [75, 76, 77, 78]. Gene expression, as an intermediate molecular phenotype closer to the phenotype, can more effectively capture the physiological state determined by the interactions between genetic variation and the environment, thus providing superior predictive ability [68]. This provides a reference for the genome‐wide prediction of other complex agronomic traits in alfalfa.

In summary, a multiomics analysis strategy was used to identify natural genome variations in alfalfa associated with salt tolerance. This research revealed numerous genetic variations that regulate gene expression across the entire genome, which can be either constitutive or conditionally significant responsive to salt stress. The salt stress‐related candidate genes identified in this study are important targets for further functional research and the exploration of superior allelic variations.

## 4 Experimental Section

### 4.1 Alfalfa Accessions and Genome Resequencing

In this study, 176 alfalfa germplasm resources that have been comprehensively evaluated in our previous studies were used [35]. These accessions originated from 45 different countries spanning Europe, Asia, and the Americas. All the samples were sequenced on the BGI platform, and ∼30 TB of raw data were obtained.

### 4.2 Variant Calling, Filtering and Annotation

Raw reads were processed using fastp (v0.23.4) to remove adapter sequences and low‐quality bases [79]. Clean reads were aligned to the Zhongmu No. 1 reference genome using BWA‐MEM (v0.7.17) with default parameters [80]. The resulting SAM files were converted to sorted BAM files using SAMtools (v1.9), and PCR duplicates were marked and removed using the markdup command [81]. For SV calling, Manta (v1.6) was executed in single‐sample mode to identify deletions and insertions. We used the default parameters and further filtered the single‐sample VCF to retain (i) calls that passed filters, (ii) those with a length of at least 50 bp and (iii) the selected variant types (deletions and insertions). We employed Manta to identify deletions and insertions separately in single samples, followed by SVimmer [82] to obtain a putative cohort‐wide consensus set. Individual‐level genotype calls within this uniformly defined discovery SV set were then refined using Graphtyper [83]. After filtering, 38,532 SVs were retained. SNPs and INDELs were called using GATK (v4.2.6.0) HaplotypeCaller with the “‐ERC GVCF” parameter [84]. Individual gVCF files were combined using CombineGVCFs, followed by joint genotyping with GenotypeGVCFs. Variant filtering was performed in two steps: (1) hard filtering using VariantFiltration with the parameters QD < 2.0, QUAL < 30.0, FS > 60.0, MQ < 40.0, and ReadPosRankSum < ‐8.0 for SNPs and QD < 2.0, MQ < 40.0, FS > 60.0, SOR > 3.0, MQRankSum < ‐12.5, and ReadPosRankSum < ‐8.0 for indels; and (2) additional filtering to remove variants with a mean sequencing depth ≤ 10, minor allele frequency < 0.05, or missing rate > 0.9. Finally, 2,821,247 high‐quality SNPs and 2,046,588 PAVs were retained and annotated using ANNOVAR [85].

### 4.3 Evaluation of Salt Stress‐Related Traits

In this study, a pot experiment was performed to evaluate the phenotypes of 176 alfalfa germplasm accessions under normal growth conditions and salt stress. Uniformly sized circular plastic pots (30 cm inner diameter, 30 cm height) lined with waterproof cloth were filled with homogenized nutrient soil. The plants were cultivated in a controlled greenhouse environment until 21 days of age, when uniformly growing plants were selected for treatment. The environmental conditions for the greenhouse experiment were 25°C ± 2°C during the day and 20°C ± 2°C at night, with 16 h of light and 8 h of darkness. The salt stress treatment involved irrigating with 200 mM NaCl solution to soil saturation and periodically replenishing to maintain the stress intensity, whereas the normal control treatment received an equal volume of distilled water to ensure comparable soil moisture. During treatment, the pots were arranged randomly and regularly rotated to minimize microenvironmental effects. After 21 days of treatment, measurements of plant height, fresh weight, and dry weight were initiated. Plant height was determined by measuring the vertical distance from the soil surface to the highest growth point of each plant using a ruler, and the average value of all the plants was taken to represent the plant height of each group. After the above‐ground parts were collected and the impurities were removed, the fresh weight was measured using an electronic balance. The samples were subsequently air‐dried in a dry, ventilated area, and the measured weight was recorded as the dry weight. For the salt tolerance index, a scale from 0∼8 was used to comprehensively assess the overall performance of each material under salt stress; each material was evaluated independently three times, and the results were averaged. The score was based on the severity of salt injury symptoms, such as leaf yellowing, wilting, and growth inhibition, where 0 indicated complete mortality and no salt tolerance, and 8 indicated no visible damage and extremely high salt tolerance. There were three biological replicates of each alfalfa germplasm, with each replicate consisting of five individual plants.

### 4.4 Genome‐Wide Association Analysis

To account for population structure and kinship, a genome‐wide association study (GWAS) was performed using EMMAX software [86]. The first three principal components (PC1‐PC3) derived from the genome‐wide SNP data were included as fixed effects in the model, based on the PCA results showing three major subpopulations among the 176 accessions. The results were visualized as Manhattan and Q‐Q plots using the R package CMplot [87]. Based on 1,000 permutation tests, the 95th percentile of the maximum −log_10_P values was 5.8, corresponding to p = 1.58 × 10^−6^. We therefore adopted ‐log_10_(P) > 6 as the genome‐wide significance threshold.

### 4.5 RNA‐Seq Analysis

A total of 176 alfalfa varieties were germinated and cultivated following the methods described in reference 35. Three biological replicates were established for each variety for phenotyping and sample collection. After 21 days of seedling growth, two replicates were subjected to salt stress treatment (200 mM NaCl), while the third replicate was maintained with normal irrigation as a control. Samples were collected on the second and fifth days after the initiation of salt stress and designated SS1 and SS2, respectively. Well‐watered control (WW) samples were collected on the same day as those in the SS1 treatment group. To minimize potential circadian influences on gene expression, SS2 samples were collected three days later at the same time of day as the SS1 samples.

For RNA‐seq, one pooled sample per variety per condition was generated and sequenced. Specifically, a five centimeter segment from the middle portion of the second leaf was collected from five individual plants, pooled, and immediately frozen in liquid nitrogen. All samples were stored at ‐80°C until RNA extraction. Total RNA was extracted using TRIzol reagent. A single sequencing library was constructed from the total RNA of each pooled sample using an Illumina TruSeq paired‐end mRNA‐Seq kit according to the manufacturer's protocol. The libraries were subsequently sequenced on an Illumina HiSeq 2500 system using TruSeq SBS v3 reagents, as described in previous studies. This yielded 528 independent sequencing libraries in total (176 varieties × 3 conditions: WW, SS1, SS2).After removing reads with low sequencing quality and those with sequencing adapters, we filtered samples with fewer than 10 million sequenced reads to prevent inaccurate quantitative analysis of unsaturated sequences. All high‐quality sequenced reads were mapped to the Zhongmu1 genome using HISAT2 (v2.0.5) [88] with default parameters.

### 4.6 Quantification of Gene Expression and Clustering Analysis

After all valid aligned reads (MQ > 20) were sorted according to their genomic positions, gene quantification was performed using featureCounts [89], and the edgeR software package was employed to identify genes that were differentially expressed between groups. For differential expression analysis, we applied TMM normalization to the raw count data, which is the standard approach for count‐based models in edgeR. Genes whose expression differed significantly between any two treatments in at least 20% of the samples were defined as differentially expressed genes (FDR < 0.05). Gene expression levels were quantified using StringTie software [90] as FPKM values, with simultaneous correction for the effects of sequencing depth and gene length. To eliminate batch effects and technical variations, the normalizeQuantiles function of the LIMMA package [91] was used to normalize the FPKM values of the expressed genes across all samples. This FPKM‐based quantile normalization ensures comparability of expression levels across samples and genotypes, and the resulting normalized values were used as input for eQTL mapping and TWAS. On the basis of the normalized data, principal component analysis was subsequently performed using the prcomp function in R (scale = TRUE), and an expression matrix heatmap was constructed using the elbow method algorithm in conjunction with the heatmap package (K = 3). After normal quantile transformation of the FPKM values for each gene, genes with normalized expression levels > 0.05 in at least 20% of the samples were selected, and finally, a mixed linear model was used for eQTL mapping analysis.

### 4.7 eQTL Mapping

eQTL mapping was performed separately for each condition (WW, SS1, and SS2) on genes expressed (FPKM > 1) in more than 20% of the accessions. GWAS was conducted using EMMAX by incorporating the kinship matrix and population structure, with the first three principal components (PC1–PC3) included as covariates. The significance threshold for eQTL detection was set to ‐log_10_(*P*) > 6, based on the same permutation results described above (GWAS section). The Benjamini–Hochberg (BH) procedure was applied for multiple testing correction. The eQTLs for a specific expression trait were identified in two steps: (1) if the physical distance between significant SNPs was < 10 kb, they were grouped into one eQTL; and (2) if the SNPs from different eQTLs were within a linkage disequilibrium (LD) block (R^2^> 0.1, calculated by Haploview), the eQTLs were merged, and the most significant SNP was retained.

### 4.8 eQTL Hotspot Identification

eQTL hotspots are genomic regions capable of influencing the expression of multiple downstream genes. To identify potential eQTL hotspot regions, we used a 1 Mb sliding window to count eQTLs across the entire genome and conducted analyses under WW, SS1, and SS2 conditions. To determine the significance threshold for eQTL hotspots, a permutation test was performed by randomly shuffling the distribution of eQTLs. During permutation, all eQTLs were assigned to random 1 Mb windows across the genome, and the number of eQTLs within each window was counted. The maximum number of eQTLs within a 1 Mb window was subsequently recorded for each permutation. After 1000 permutations, on the basis of the distribution of the maximum eQTL counts obtained from the permutations, the significance threshold was set to 98 eQTLs/Mb (*p* < 0.01). Any window containing a number of eQTLs exceeding this threshold was considered a potential distal eQTL hotspot regulatory region. Overlapping or adjacent hotspot regions were merged into a single region.

### 4.9 XGBoost Regression Model Construction

FPKM expression matrices derived from RNA‐seq analysis under well‐watered (WW), salt stress 1 (SS1), and salt stress 2 (SS2) conditions, together with a set of 1,916 identified transcription factors (TFs), were used to construct XGBoost regression models [92]. Model construction was performed using the Python machine‐learning libraries scikit‑learn and XGBoost [93]. For each eGene, TF expression levels were used as predictors and eGene expression was used as the response variable. The model was trained with the squared‐error regression objective (reg:squarederror), learning rate eta = 0.1, maximum tree depth max_depth = 3, subsampling rate subsample = 0.8, L1 regularization reg_alpha = 0, L2 regularization reg_lambda = 1, nrounds = 50, and nthread = 4. Five‐fold cross‐validation with RMSE as the evaluation metric and early stopping after 10 rounds was used to evaluate model performance. For feature importance, we extracted gain‐based importance from the trained XGBoost model. For each eGene, TFs were ranked according to Gain, which measures the relative contribution of each TF to reducing prediction error across tree splits. The Gain values were further normalized by the maximum Gain within each eGene model, and the top three TFs for each eGene were retained for network construction.

### 4.10 Transcriptome‐Wide Association Study

The TWAS was performed using GAPIT software (v3.0) [94]. Specifically, the analysis employed the compressed mixed linear model (CMLM) implemented in the software as the core statistical model. During the modeling process, to control for potential false positive associations caused by population structure, the first three principal components of genetic variation calculated by the GCTA tool were included as covariates in the model [95]. With respect to significance determination, an independent statistical significance threshold was set for each phenotypic trait under normal and salt stress conditions. The *p* value corresponding to this threshold was derived by controlling the false discovery rate (FDR) at 0.05 using the Benjamini‒Hochberg procedure.

### 4.11 Mendelian Randomization (MR) Test

Here, we employed the 2SLS framework from the maize drought study [17, 96], for exploratory gene prioritization. For each gene, we screened all eQTLs with ‐log_10_(P) > 5 for gene expression as candidate instruments; this threshold was chosen because stricter cutoffs (e.g., ‐log_10_(P) > 6 as used for primary eQTL mapping) yielded too few SNPs per gene for reliable MR screening. The strongest MR association per gene was retained, and a significance threshold of ‐log10(P_MR)>5 was applied for candidate prioritization.

### 4.12 Genome‐Wide Prediction of Traits Related to Salt Stress in Alfalfa

The prediction methods used in this study included primarily GTBLUP (transcriptome‐assisted GBLUP) and DNNGP [75, 97]. A repeated 10‐fold cross‐validation framework was employed to evaluate model performance. For the GBLUP model prediction, SNP sets were selected on the basis of different thresholds (*p* < 1.0 × 10^−^^4^, *p* < 1.0 × 10^−^^3^, and *p* < 1.0 × 10^−^^2^), including all the markers significantly associated with each trait. As a transcriptome‐assisted linear mixed‐model benchmark, GTBLUP was performed using the BGLR package in R. When the DNNGP model was used for genome‐wide prediction, genes associated with the trait obtained from the TWAS or MR test were ranked by significance. The top 500 genes for each trait were selected, with their expression uniformly measured at the SS2 stage. The specific parameters for the DNNGP model were ″–batch_size 28 –lr 0.01 –epoch 100 –dropout1 0.5 –dropout2 0.3 –patience 5 –cv 10 –part 1 –earlystopping 10.

### 4.13 Generation and Functional Analysis of MsRD26 Overexpression Lines

MsRD26 was amplified from cDNA of ‘Zhongmu No. 1’ alfalfa using specific primers and cloned into the plant binary overexpression vector pCAMBIA1300. The construct was introduced into ‘Zhongmu No. 4’ leaf explants via Agrobacterium tumefaciens EHA105‐mediated transformation, followed by hygromycin‐based selection and plant regeneration. Putative transgenic plants were initially screened by PCR with 35S promoter‐specific primers. RT‐qPCR was then performed to confirm MsRD26 transcript levels and, additionally, to examine the expression of key ABA pathway‐related genes. For this, total RNA was extracted from leaf tissues at 0 and 12 h after 200 mM NaCl treatment. First‐strand cDNA was synthesized from 1 µg of total RNA using the HiScript III first Strand cDNA Synthesis Kit (Vazyme Biotech, Nanjing, China) following the manufacturer's instructions (including genomic DNA elimination and reverse transcription with oligo(dT) primers). Quantitative PCR was performed using SYBR Premix Ex Taq (TaKaRa, Japan) on a 7500 Real‐Time PCR System (Applied Biosystems) with three technical replicates per biological replicate. The alfalfa *UBL‐2a* gene served as the internal control, and relative gene expression levels were calculated using the 2^−ΔΔCt^ method [98]. All primers are listed in Table S10, and gene IDs corresponding to the studied genes are given in Table S11. Finally, uniformly grown wild‐type and overexpression lines were subjected to 200 mM NaCl stress, and phenotypic differences were compared to verify the role of *MsRD26* in salt tolerance.

### 4.14 Electrophoretic Mobility Shift Assay (EMSA)

The coding sequence of *MsRD26* was cloned into the pCold vector with a 6×His tag and expressed as a recombinant protein (36.3 kDa) in E. coli. For double‐stranded DNA probe preparation, equal amounts of biotin‐labeled forward and reverse oligonucleotides for Abf2‐1 (aagagcgtgttgtttacgtatgtttttttatttta) and its mutant version Abf2‐1‐mut (aagagcgtgttTtttTTTtTtgtttttttatttta, with mutated bases shown in uppercase) were separately mixed, heated at 97 °C for 2 min, and then slowly cooled to room temperature for annealing. For competition assays, a 50‐fold molar excess of unlabeled Abf2‐1 probe (cold competitor) was added. The MsRD26 recombinant protein was incubated with biotin‐labeled probes at room temperature for 20 min in binding buffer using a chemiluminescent EMSA kit (Mich Scientific, MC702). The reaction mixtures were separated by nondenaturing electrophoresis on 6 % native polyacrylamide gels in 0.5× TBE buffer at 100 V until the dye front reached two‐thirds of the gel length. The DNA‐protein complexes were then transferred to a nylon membrane in 0.5× TBE at 100 V for 1 h, followed by UV cross‐linking. The biotin‐labeled probes were detected using a chemiluminescent nucleic acid detection module according to the manufacturer's instructions (Mich Scientific, MC702), and signals were visualized using a chemiluminescence imaging system (Shanghai Qinxiang, ChemiScope 6100). As a positive control, a positive protein and positive probe were included in the assay.

### 4.15 Dual‐Luciferase Reporter Assay

The promoter sequence of *ABF2* was amplified and cloned into the pGreenII 0800–35Smini‐LUC vector as the reporter construct, with Renilla luciferase (REN) as an internal control. The coding sequence of MsRD26 was cloned into the pCambia1300‐221‐Flag vector as the effector construct. Both constructs were transformed into Agrobacterium tumefaciens strain GV3101. For transient expression assays, Agrobacterium cultures carrying the reporter and effector constructs were resuspended in infiltration buffer (10 mM MES, 0.02 mM acetosyringone) to an appropriate optical density. The mixtures were co‐infiltrated into the abaxial surface of Nicotiana benthamiana leaves using needleless syringes. After 48 h of infiltration, the infiltrated leaf discs were collected for analysis. For LUC imaging, leaf samples were sprayed with D‐luciferin substrate (Promega, E1605), incubated for 5–10 min, and then imaged using a NightSHADE LB 985 imaging system (Berthold). For quantitative measurement, leaf tissues were homogenized in lysis buffer, and firefly luciferase (LUC) and Renilla luciferase (REN) activities were measured sequentially using the Dual‐Luciferase Reporter Assay System (Promega, E1910) on a NightSHADE LB 100 microplate reader (Berthold). The relative promoter activity was calculated as the ratio of LUC to REN activity. At least three biological replicates were included for each construct combination.

### 4.16 Statistical Analysis

All statistical analyses were performed using R version 4.2.1 and Python 3.9. Data are presented as mean ± standard error (SE) for bar charts, while box plots display the median and interquartile range. Sample sizes (n) are provided in each figure legend. For comparisons between two groups, two‐sided Student's t‐test was applied. All statistical tests were two‐sided.

## Author Contributions

L.C., J.K. and Q.Y conceived and designed the research. L.C., Y.Z., X.M., J.B., F.H., H.L., Z.X., X.W., T.Z., R.L., J.K., and L.Z. performed the experiments. L.C., Y.Z., X.M., and J.B. analyzed the data. L.C. and Y.Z. wrote the manuscript. All the authors read and approved the final manuscript.

## Funding

This work was supported by the National Natural Science Foundation of China (32441018 and 32371757) and the major demonstration project “The Open Competition” for Seed Industry Science and Technology Innovation in Inner Mongolia (No. 2022JBGS0016).

## Conflicts of Interest

The authors declare that they have no conflicts of interest.

## Supporting information




**Supporting File 1**: advs77098‐sup‐0001‐SuppMat.docx.

**Supporting File 2**:: advs77098‐sup‐0002‐TableS1.xlsx.

**Supporting File 3**: advs77098‐sup‐0003‐TableS2.xls.

**Supporting File 4**: advs77098‐sup‐0004‐TableS3.xlsx.

**Supporting File 5**: advs77098‐sup‐0005‐TableS4.xlsx.

**Supporting File 6**: advs77098‐sup‐0006‐TableS5.xlsx.

**Supporting File 7**: advs77098‐sup‐0007‐TableS6.xlsx.

**Supporting File 8**: advs77098‐sup‐0008‐TableS7.xlsx.

**Supporting File 9**: advs77098‐sup‐0009‐TableS8.xlsx.

**Supporting File 10** advs77098‐sup‐0010‐TableS9.xlsx.

**Supporting File 11**: advs77098‐sup‐0011‐TableS10.xlsx.

**Supporting File 12**: advs77098‐sup‐0012‐TableS11.xlsx.

**Supporting File 13**: advs77098‐sup‐0013‐TableS12.xlsx.

## Data Availability

The genome resequencing data for the 176 alfalfa accessions have been deposited in the NCBI Sequence Read Archive (SRA) under BioProject PRJNA1197171. The raw RNA‐seq data (528 libraries) have been deposited in the Genome Sequence Archive (GSA) at the China National Center for Bioinformation (CNCB) under accession number CRA074515. All supplementary tables are available in the online Supporting Information. The data that support the findings of this study are openly available in the above repositories.
