# Supplementary material for: Conditionally Significant eQTL and TWAS Analyses Identify MsRD26 as a Major Candidate Positive Regulator of Salt Tolerance in Medicago sativa L
Source: Adv Sci (Weinh). 2026 Aug 11;13(61):e77098. doi: 10.1002/advs.77098 (PMC13460419; doi:10.1002/advs.77098)
Supplement: Supplementary file 1 [file ADVS-13-e77098-s006.docx]

**Supplementary Information**

**Dynamic eQTL and Transcriptome-Wide Association Analyses Reveal MsRD26 as a Key Regulator of Salt Tolerance in Alfalfa**

*Lin Chen*^1,3^*, *Yuqi Zhang*^1,3^, *Xinyue Ma*^1,3^, *Jinpeng Bi*^2,3^, *Fei He*^1^, *Li Zhao*^1^, *Haiyue Lei*^1^, *Zhengqin* *Xiao*^1^, *Xue Wang*^1^, *Tiejun Zhang*^1^, *Ruicai Long*^1^, *Junmei Kang*^1^*, *Qingchuan Yang*^1^*

^1^Institute of Animal Science, Chinese Academy of Agricultural Sciences, Beijing 100193, China

^2^State Key Laboratory of Vegetable Biobreeding, Institute of Vegetables and Flowers, Chinese Academy of Agricultural Sciences, Beijing 100193, China

*Correspondence: Lin Chen: [chenlin@caas.cn](mailto:chenlin@caas.cn)

Junmei Kang: kangjunmei@caas.cn

Qingchuan Yang: yangqingchuan@caas.cn

^3^Lin Chen, Yuqi Zhang, Xinyue Ma and Jinpeng Bi contributed equally to this work.

**Table of Content**

[Figure S1. Distribution of SNPs and PAVs across the entire genome and population structure analysis. .	3](#_Toc1701)

[Figure S2. Manhattan and Q‒Q plots of PH, FW and DW under normal conditions and salt stress. A, PH. B, FW. C, DW.	4](#_Toc32474)

[Figure S3. GO analysis of the DEGs. .	5](#_Toc8527)

[Figure S4. Expression patterns of ABA-related genes. .	6](#_Toc26146)

[Figure S5. Overlap of the GWAS-identified genes and differentially expressed genes (DEGs). .	7](#_Toc27331)

[Figure S6. Characteristics of the eQTLs and their associated eGenes.	7](#_Toc7298)

[Figure S7. Overlapping eGenes regulated by local eQTLs and distant eQTLs under WW, SS1 and SS2 conditions.	8](#_Toc23947)

[Figure S8. Statistical analysis of static local and dynamic local eQTLs and their corresponding eGenes under WW, SS1 and SS2 conditions.	9](#_Toc6160)

[Figure S9. Transcription factor families and GO terms enriched in eGenes regulated only by dynamic local eQTLs under salt stress	10](#_Toc11859)

[Figure S10. Manhattan plot of TWAS for DW, FW, and PH under WW, SS1 and SS2 conditions.	10](#_Toc7689)

[Figure S11. The genes identified by TWAS and TF enrichment..	11](#_Toc27545)

[Figure S12. Analysis of the correlations between the expression of MsGolS1, MsOPR1, and MsHAK5 under SS1 or SS2 conditions and the DWsalt/ck, FWsalt/ck, and PHsalt/ck traits and the salt tolerance index.	12](#_Toc20546)

[Figure S13. MR tests to determine the associations between gene expression and PH, DW, and FW under salt stress.	13](#_Toc4075)

[Figure S14. Allelic comparison of the expression levels of MsNAC047 (A), MsMYB15 (B), and MsOPR1 (C) and salt stress-related traits on the basis of the lead SNPs of eQTLs..	14](#_Toc18167)

[Figure S15. Relative expression levels of MsRD26 in the WT and OE lines.	15](#_Toc17133)

[Figure S16. Phenotype comparison of the WT and OE lines under salt stress.	15](#_Toc8747)

[Figure S17. Superior alleles analysis of the Top25 and Bottom25 accessions based on DWsalt/ck, FWsalt/ck, and PHsalt/ck.	16](#_Toc10177)

[Figure S18.The expression of NAGs and PAGs associated with DWsalt/ck, FWsalt/ck, and PHsalt/ck identified by TWAS..	16](#_Toc5884)

[Figure S19. Genome-wide prediction of salt tolerance-related traits using significant SNPs and TWAS/MR-identified gene expression.	18](#_Toc19338)

[Figure S20. MsRD26 binds to the MsABF2 promoter and activates the expression of ABA signaling pathway-related genes.	19](#_Toc24719)

Figure S1. Distribution of SNPs and PAVs across the entire genome and population structure analysis. A-B, Distribution of SNPs and PAVs. C, Population structure of the 176 alfalfa accessions.

Figure S2. Manhattan and Q‒Q plots of PH, FW and DW under normal conditions and salt stress. A, PH. B, FW. C, DW.

Figure S3. GO analysis of the DEGs. A, The significant biological pathways associated with salt-repressed DEGs. B, The significant biological pathways associated with salt-inducible DEGs. C, The significant biological pathways associated with salt response-related genes.

Figure S4. Expression patterns of ABA-related genes. A, The expression of 15 ABA-related genes repressed by salt. B, The expression of 18 ABA-related genes was induced by salt. C, The expression of 27 ABA-related genes varied in response to salt.

Figure S5. Overlap of the GWAS-identified genes and differentially expressed genes (DEGs). A, Venn diagram showing the number of overlapping genes identified by GWAS and the DEGs. B, Expression patterns of these overlapping genes.

Figure S6. Characteristics of the eQTLs and their associated eGenes. A, Number of eGenes associated with one or more eQTLs. The x-axis represents the number of eQTLs mapped for each eGene, and the y-axis denotes the number of eGenes within each group. B, Distribution of local eQTLs relative to the transcription start sites (TSSs) of the regulated eGenes.

Figure S7. Overlapping eGenes regulated by local eQTLs and distant eQTLs under WW, SS1 and SS2 conditions.

Figure S8. Statistical analysis of static local and dynamic local eQTLs and their corresponding eGenes under WW, SS1 and SS2 conditions. A, Number of static local and dynamic local eQTLs identified under the three conditions. B, The corresponding eGenes regulated by these eQTLs. C, Overlapping and unique eGenes regulated by the dynamic local eQTLs under WW, SS1 and SS2 conditions.

Figure S9. Transcription factor families and GO terms enriched in eGenes regulated only by dynamic local eQTLs under salt stress. A, TF families. B, Biological pathways.

Figure S10. Manhattan plot of TWAS for DW, FW, and PH under WW, SS1 and SS2 conditions. The genes reported to be related to abiotic stress in alfalfa and other plants are marked.

Figure S11. The genes identified by TWAS and TF enrichment. A, Venn diagram illustrating the numbers of overlapping and unique PAGs and NAGs identified under the SS1 and SS2 conditions for all the salt stress-related traits. B, TF families enriched in these genes identified by TWAS for DW_salt/ck_, FW_salt/ck_, PH_salt/ck_ and the salt tolerance index.

Figure S12. Analysis of the correlations between the expression of MsGolS1, MsOPR1, and MsHAK5 under SS1 or SS2 conditions and the DWsalt/ck, FWsalt/ck, and PHsalt/ck traits and the salt tolerance index. A, MsGolS1. B, MsOPR1. C, MsHAK5.

Figure S13. MR tests to determine the associations between gene expression and PH, DW, and FW under salt stress.

Figure S14. Allelic comparison of the expression levels of MsNAC047 (A), MsMYB15 (B), and MsOPR1 (C) and salt stress-related traits on the basis of the lead SNPs of eQTLs. For A，the sample sizes for the AA and AT genotype groups are n = 102 and n = 41, respectively. For B，the sample sizes for the TT and TC genotype groups are n = 108 and n = 39, respectively. For C，the sample sizes for the AA and AG genotype groups are n = 87 and n = 66, respectively. Statistical significance was determined by two-sided Student's t-test. P values are shown in the figure.

Figure S15. Relative expression levels of MsRD26 in the WT and OE lines. Data are presented as means ± SE (n = 3 biological replicates per group). Statistical significance was determined by Student's t-test. ns indicates no significant difference, * indicates significant difference at P < 0.05, ** indicates significant difference at P < 0.01, *** indicates significant difference at P < 0.001.

Figure S16. Phenotype comparison of the WT and OE lines under salt stress. A, Stems of the WT and OE lines under normal and salt stress conditions. B-C, Statistical analysis of POD activity and SOD activity in the WT and OE lines under normal and salt stress conditions. Data are presented as means ± SE (n = 3 biological replicates per group). Statistical significance was determined by Student's t-test. ns indicates no significant difference, * indicates significant difference at P < 0.05, ** indicates significant difference at P < 0.01.

Figure S17. Superior alleles analysis of the Top25 and Bottom25 accessions based on DWsalt/ck, FWsalt/ck, and PHsalt/ck. A-C, DWsalt/ck. D-F, FWsalt/ck. G-I, PHsalt/ck. For A, D, G, Distribution of superior alleles between the top 25 salt-tolerant materials (Top25) and the bottom 25 salt-tolerant materials (Bottom25). Among them, gray boxes represent unfavorable alleles, white boxes represent missing alleles, and other colors boxes represent superior alleles. B,E,H, Correlations between the number of superior alleles and DWsalt/ck, FWsalt/ck, and PHsalt/ck, respectively. C, F, I, Comparison of the number of superior alleles between Top25 and Bottom25. n = 25 accessions per group. Statistical significance was determined by two-sided Student's t-test. P values are shown in the figure.

Figure S18. The expression of NAGs and PAGs associated with DWsalt/ck, FWsalt/ck, and PHsalt/ck identified by TWAS. A-B, DWsalt/ck. C-D, FWsalt/ck. E-F, PHsalt/ck. A,C,E, Heatmap showing the expression patterns of NAG and PAG genes associated with DWsalt/ck, FWsalt/ck, and PHsalt/ck, respectively, identified by the TWAS. Each column represents a gene, and each row represents a variety. B, D, F, Comparison of the expression of NAGs and PAGs among Top25 and Bottom25 under the SS2 condition. Beeswarm plots showing the distribution of average expression values of NAGs (144 genes,154 genes, 166 genes for DWsalt/ck, FWsalt/ck, and PHsalt/ck, respectively) and PAGs (167 genes, 153 genes, 209 genes for DWsalt/ck, FWsalt/ck, and PHsalt/ck, respectively) in Top25 and Bottom25 accessions under SS2 conditions. Each point represents an individual gene. For each accession, the mean expression level of all NAGs or PAGs was calculated. Statistical significance between Top25 and Bottom25 groups was assessed by two-sided Student's t-test. P values are shown in the figure.

Figure S19. Genome-wide prediction of salt tolerance-related traits using significant SNPs and TWAS/MR-identified gene expression. Genome-wide predictions for DWsalt/ck, FWsalt/ck, and PHsalt/ck by using significant SNPs at different thresholds and the expression of the genes identified by TWAS or the MR test. Genome-wide predictions of the SNPs were conducted by the GBLUP model. Genome-wide predictions of gene expression were conducted by the DNNGP model.

Figure S20. MsRD26 binds to the MsABF2 promoter and activates the expression of ABA signaling pathway-related genes. A, Relative expression levels of ABA signaling pathway-related genes in MsRD26 overexpression transgenic lines. Compared to the wild type (WT), the expression levels of MsNCED3, MsABI1, and MsABF2 were significantly upregulated in the overexpression lines (OE1, OE2, OE4), indicates significant difference compared to WT (P<0.01). Error bars represent standard error (SE). Data are presented as means ± SE (n = 3 biological replicates per group). Statistical significance was determined by Student's t-test. ** indicates significant difference at P < 0.01. B, Electrophoretic mobility shift assay (EMSA) detecting the binding of MsRD26 to the MsABF2 promoter. Left panel shows the wild-type (ABF2-wild) and mutant (ABF2-mut) probe sequences in the MsABF2 promoter region (-1174 to -1140), with the predicted MsRD26 binding motif highlighted in red. Right panel shows that MsRD26 can form a shifted band with the biotin-labeled wild-type ABF2 probe; this binding is abolished by competition with unlabeled cold probe or by the mutant probe, indicating specific binding of MsRD26 to this motif. C, Schematic diagram of the dual-luciferase reporter system constructs. The Reporter vector contains the LUC reporter gene driven by proMsABF2 and the REN internal reference gene driven by 35S. The Effector vector contains the MsRD26 gene or empty vector control (Control) driven by the Ubi promoter. D, Chemiluminescence imaging results of the dual-luciferase reporter assay in Nicotiana benthamiana leaves. 1: 1300EV + 800-proMsABF2 (control); 2: 1300-MsRD26 + 800-proMsABF2 (experimental group). The color scale indicates luminescence intensity (CPS). E, Quantitative analysis of the dual-luciferase reporter assay. Co-expression of MsRD26 significantly enhanced the luciferase activity driven by proMsABF2 compared to the control group (n=4, P=1.28E-04). Error bars represent standard error (SE).

Table S1. GWAS results for salt-related traits

Table S2. Statistical analysis of the RNA-seq data

Table S3. Genes in common between the GWAS and DEGs

Table S4. eQTLs identified in this study

Table S5. eQTL hotspots for distant eQTLs

Table S6. TFs and corresponding eGenes

Table S7. Genes associated with salt stress identified by TWAS

Table S8. Genes associated with salt stress identified by MR tests

Table S9. List of DEGs between the WT and OE4 lines under salt stress

Table S10. The primers used in this study

Table S11. The gene names used in this study

Table S12. Superior allele frequency differences between the bottom and top 25% accessions across multiple sites and traits, assessed by Fisher's exact test.
